# Supplementary figures and images for: Quantitative PCR from human genomic DNA: The determination of gene copy numbers for congenital adrenal hyperplasia and RCCX copy number variation
Source: PLoS One. 2022 Dec 1;17(12):e0277299. doi: 10.1371/journal.pone.0277299 (PMC9714944; doi:10.1371/journal.pone.0277299)

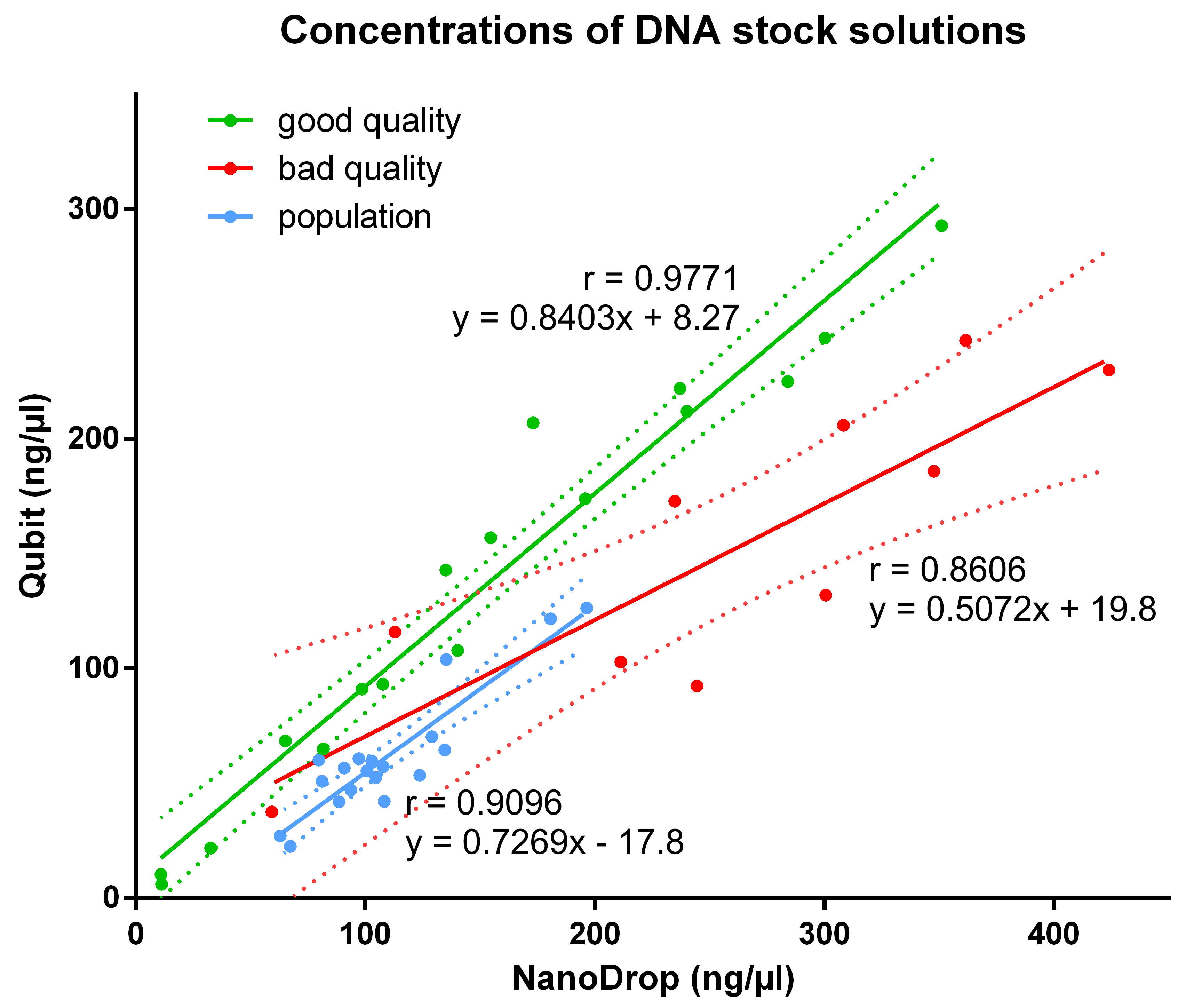

Supplement: S1 Fig — Pearson’s correlation coefficient is indicated by “r”. The data of one predefined dilution (first of separately diluted ones) of each sample for positive control and calibration curve was included in the study groups. Repeatability values of DNA concentration determinations based on positive control samples were 3.93 CV% for NanoDrop and 2.58 CV% for Qubit, and reproducibility values were 3.99 CV% and 4.22 CV%, respectively. The correlation of all samples between NanoDrop and Qubit was high (Pearson’s r = 0.887, p<0.0001), although the concentrations of DNA stock solutions measured by Qubit were significantly lower (Wilcoxon test: p<0.0001). Pearson correlation coefficients (r) between NanoDrop and Qubit values in the “bad quality” study group suggest that DNA quality influenced DNA concentration measurements. (TIF) [file pone.0277299.s001.tif]

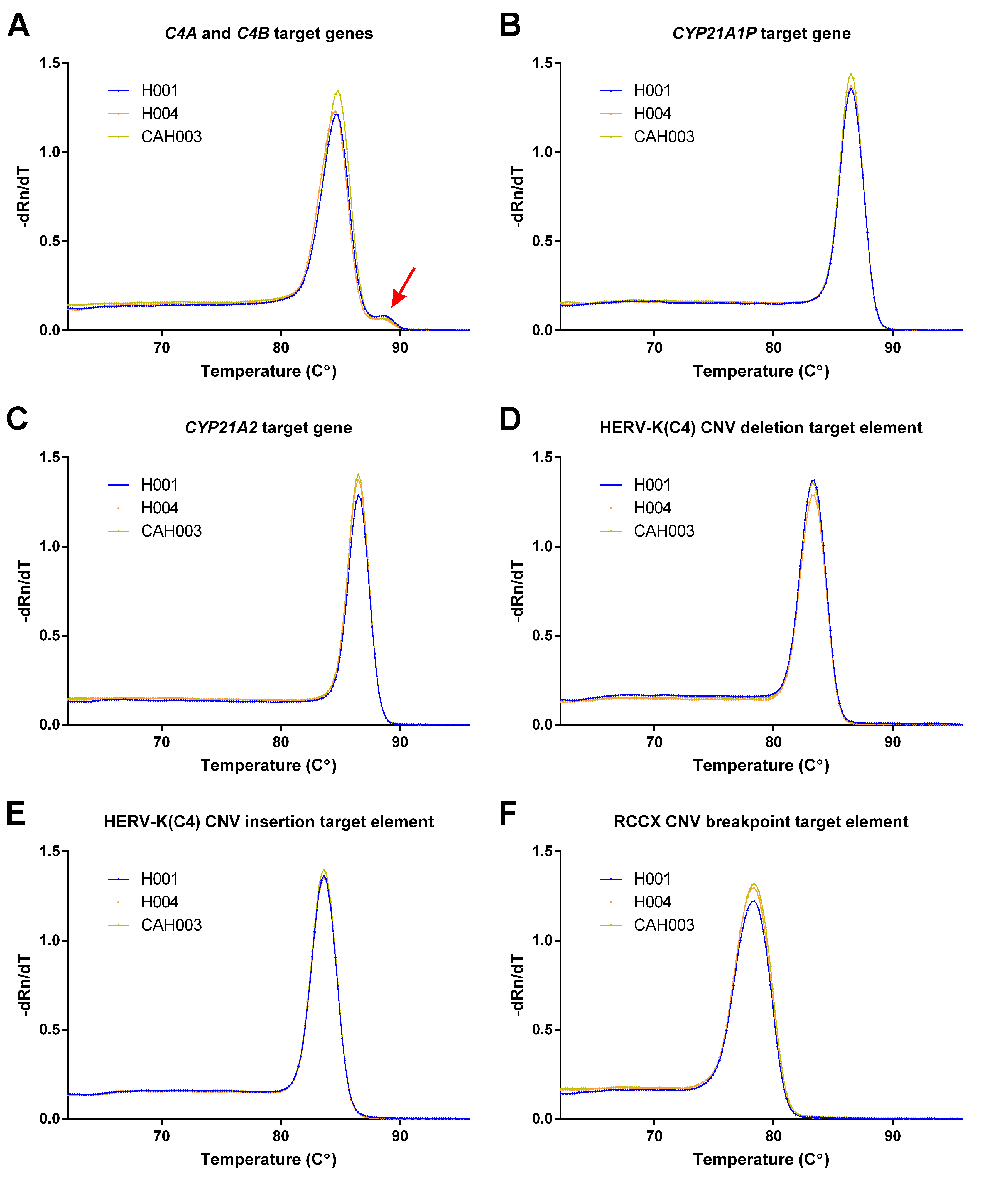

Supplement: S2 Fig — Rn is the normalized fluorescence of the reporter dye. Red arrow shows the peak of a non-specific product. (TIF) [file pone.0277299.s002.tif]

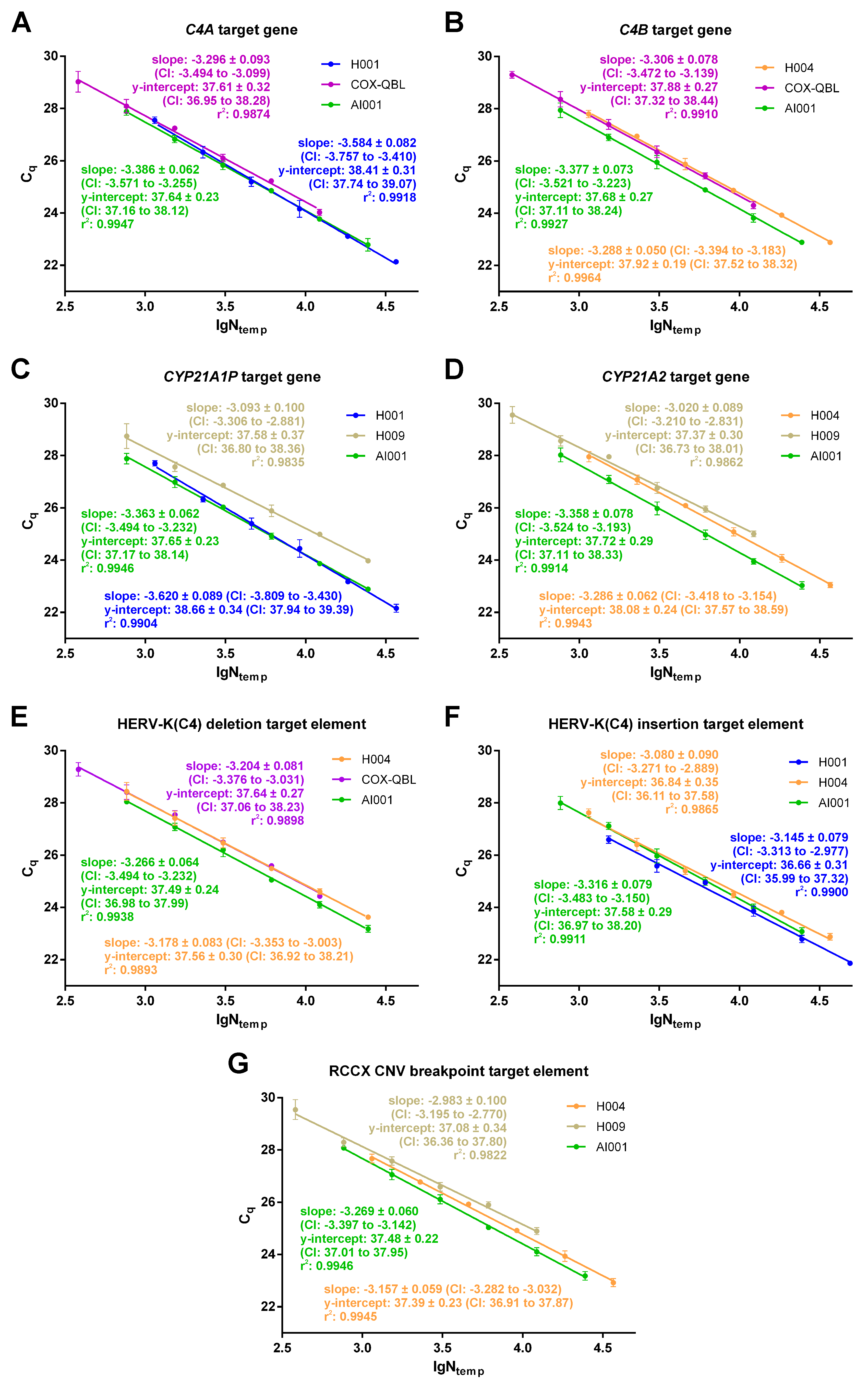

Supplement: S4 Fig — Ntemp is the genomic copy number of the particular target genetic elements, which is conditional on the amount of genomic DNA in the series of dilutions (2.5, 5, 10, 20, 40 and 80 ng total DNA in a measurement) and the gene copy number of target elements in the diploid genome (1–4). CI means 95% confidence interval. The CIs of the lines are not depicted because they would be too close the lines to discern. (TIF) [file pone.0277299.s004.tif]

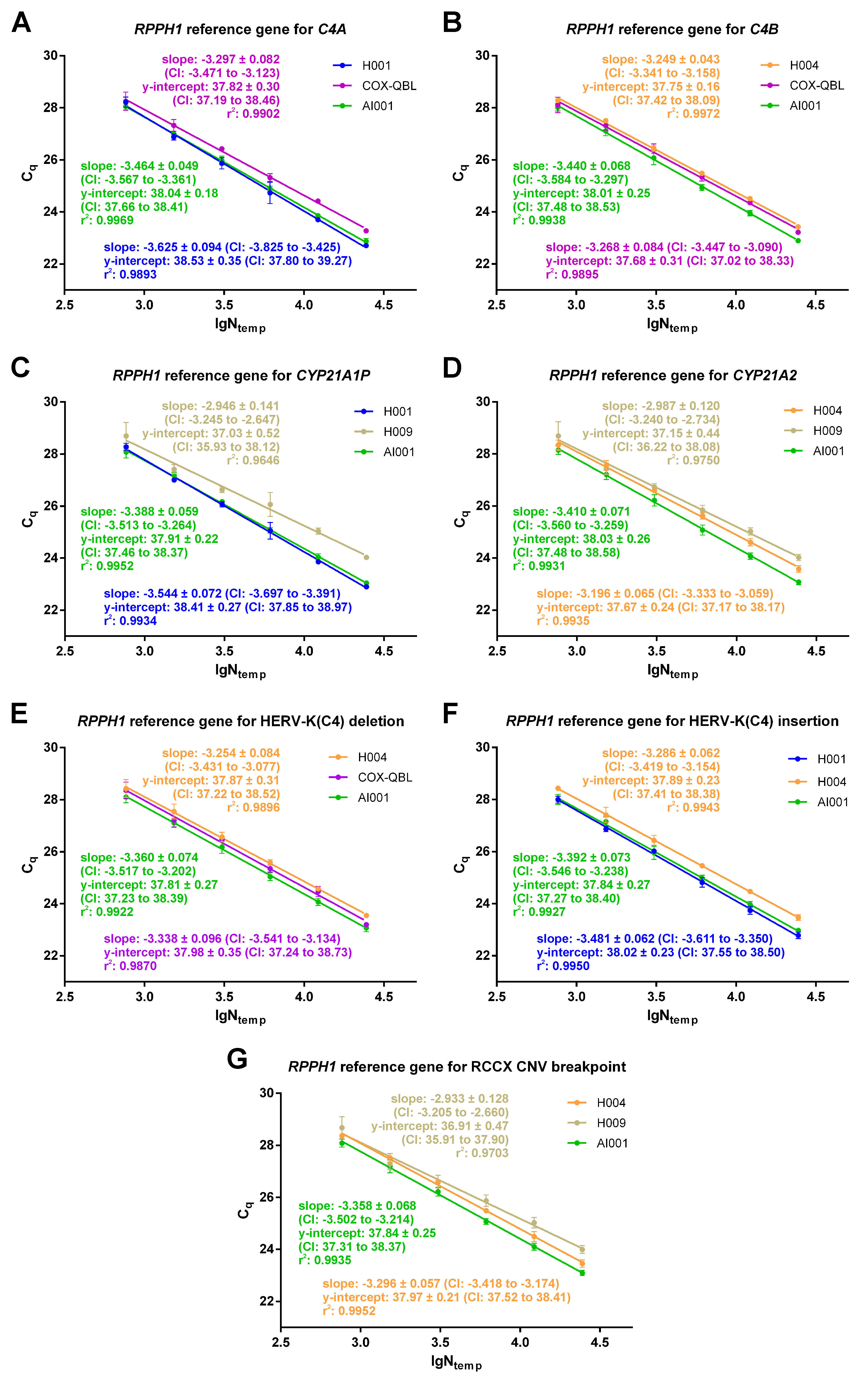

Supplement: S5 Fig — Ntemp is the genomic copy number of the target genetic elements of the RPPH1 gene, which is conditional on the amount of genomic DNA in the series of dilutions (2.5, 5, 10, 20, 40 and 80 ng total DNA in a measurement). CI means 95% confidence interval. The CIs of the lines are not depicted because they would be too close the lines to discern. (TIF) [file pone.0277299.s005.tif]

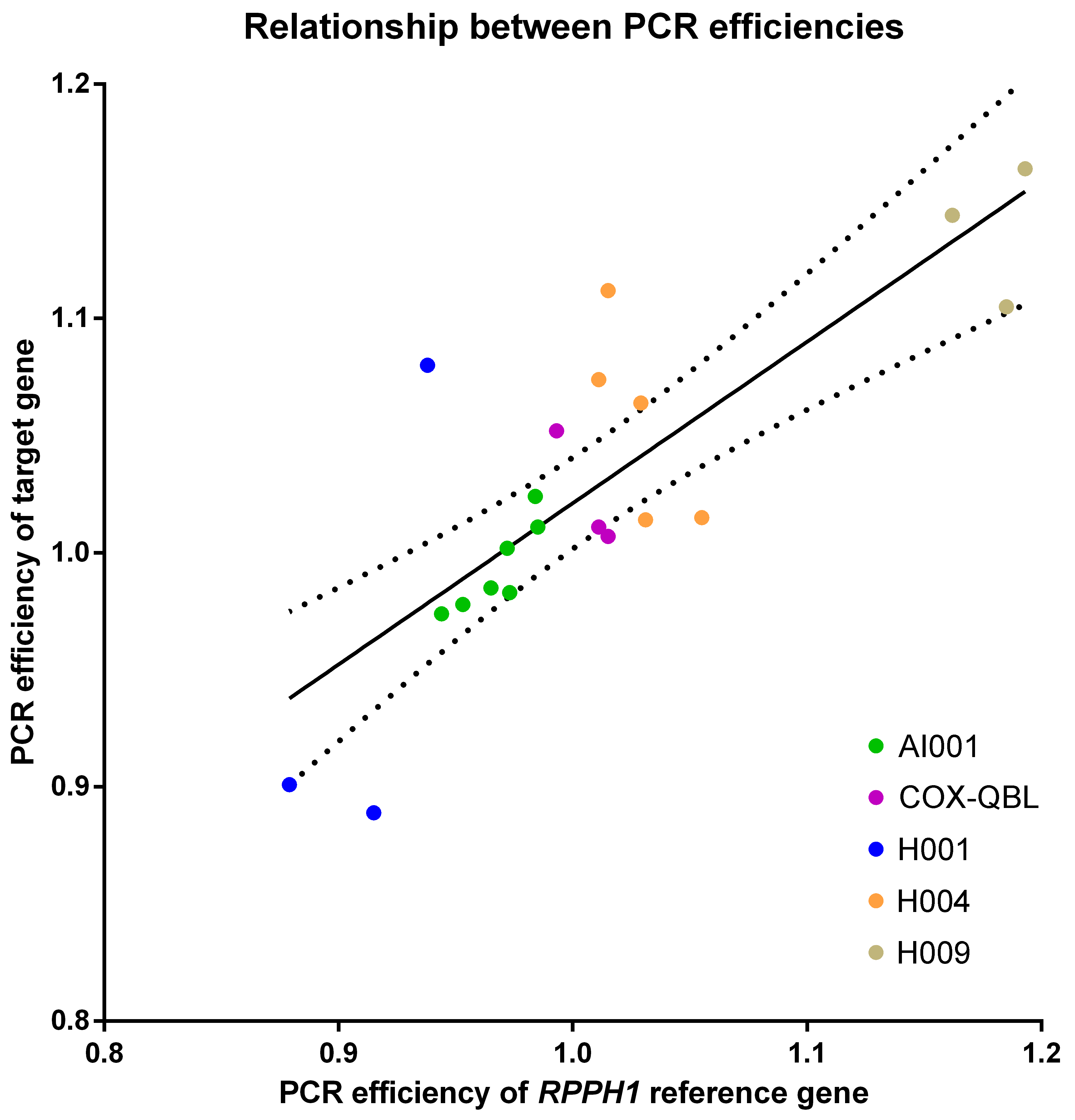

Supplement: S6 Fig — Black line is a simple linear regression. Dotted line indicates the 95% confidence intervals. (TIF) [file pone.0277299.s006.tif]

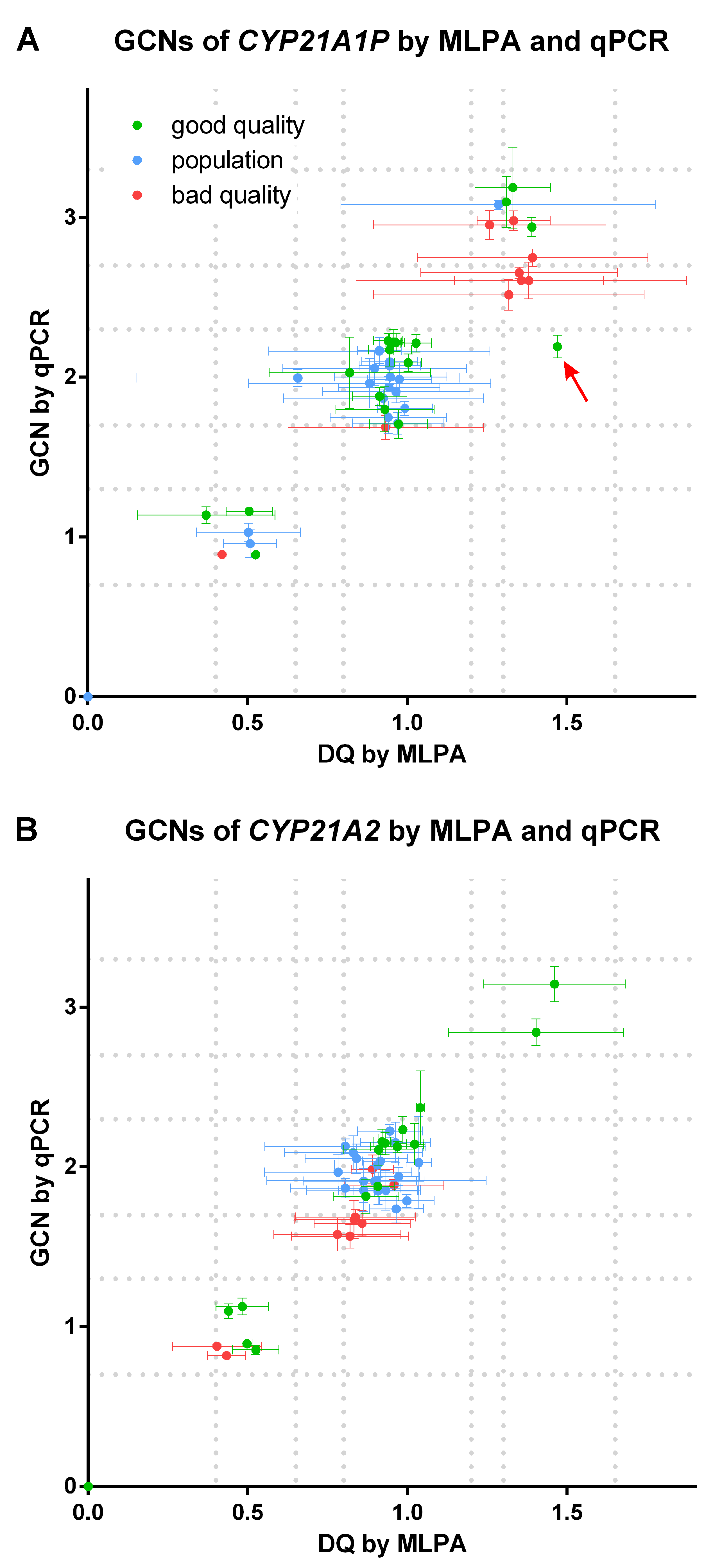

Supplement: S7 Fig — Ambiguity thresholds (±0.3 of integer GCNs for qPCR and according to the manual for MLPA) are indicated by grey dotted lines. Bars indicate the standard deviation of GCNs in the case of qPCR, and the standard deviation of the dosage quotients (equivalent of GCN in MLPA) of different probes for the same CYP21 gene in the case of MLPA. (TIF) [file pone.0277299.s007.tif]

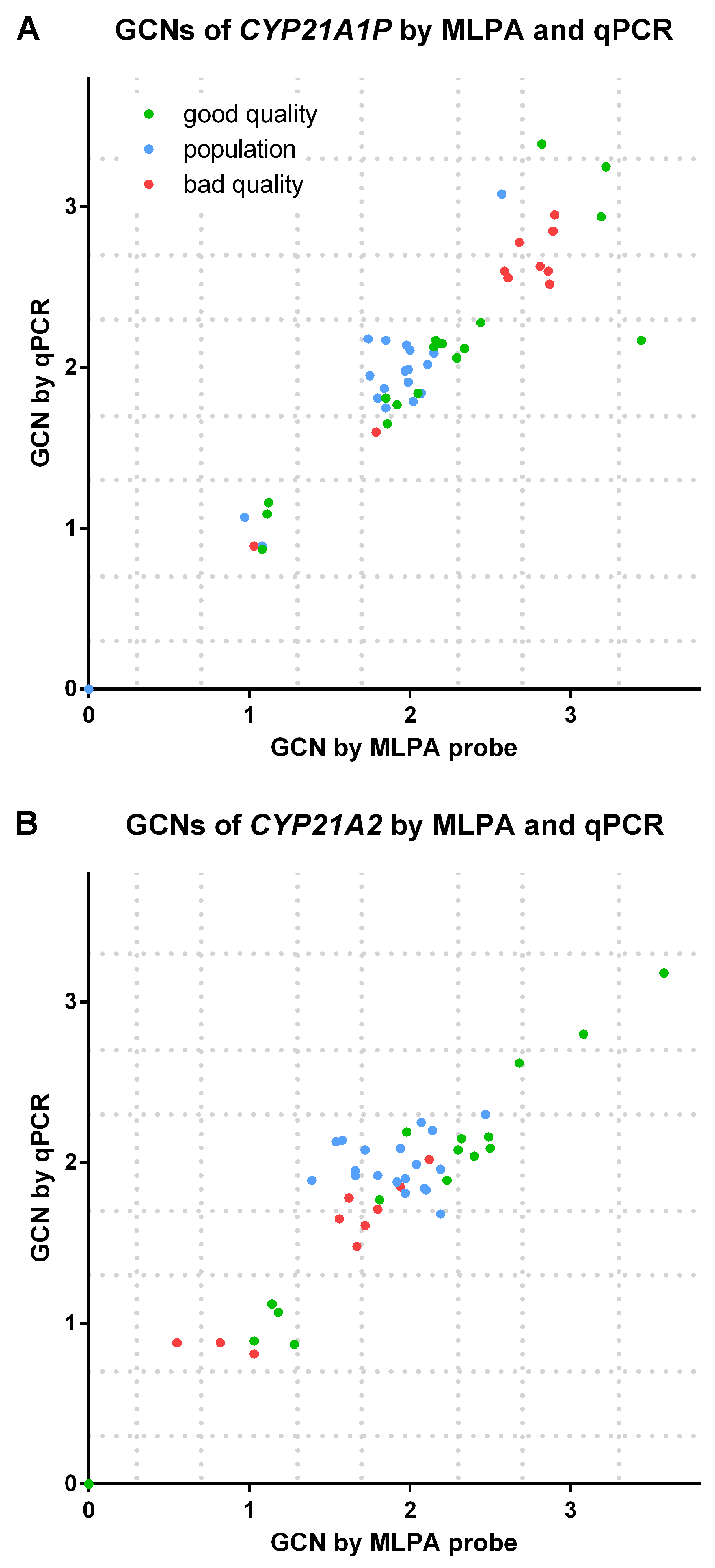

Supplement: S8 Fig — Two MLPA hybridization probes (15221-L20261 and 15221-L20262) detect the two alleles of the same 8 bp deletion variant in CYP21 genes, which is also detected by CYP21A1P and CYP21A2 qPCR assays in the current study. Therefore, these probes and assays are suitable for direct comparison after normalization with an internal reference probe. The MLPA reference probe 16316-L21434 was used for the normalization of the CYP21A2 probe (15221-L20261) and CYP21A1P probe (15221-L20262), because this reference probe showed the highest correlation with the CYP21 probes and the other MLPA reference probes. Only the GCNs of the replicates of the first replicate measurement were used for qPCR, because MLPA according to the official manual applies to one measurement of each DNA sample by default. The ratios of CYP21 and reference probes of MLPA were tuned to approximately zero average relative error, as was done for qPCR. Ambiguity thresholds (±0.3 of integer GCNs) are indicated by grey dotted lines. (TIF) [file pone.0277299.s008.tif]

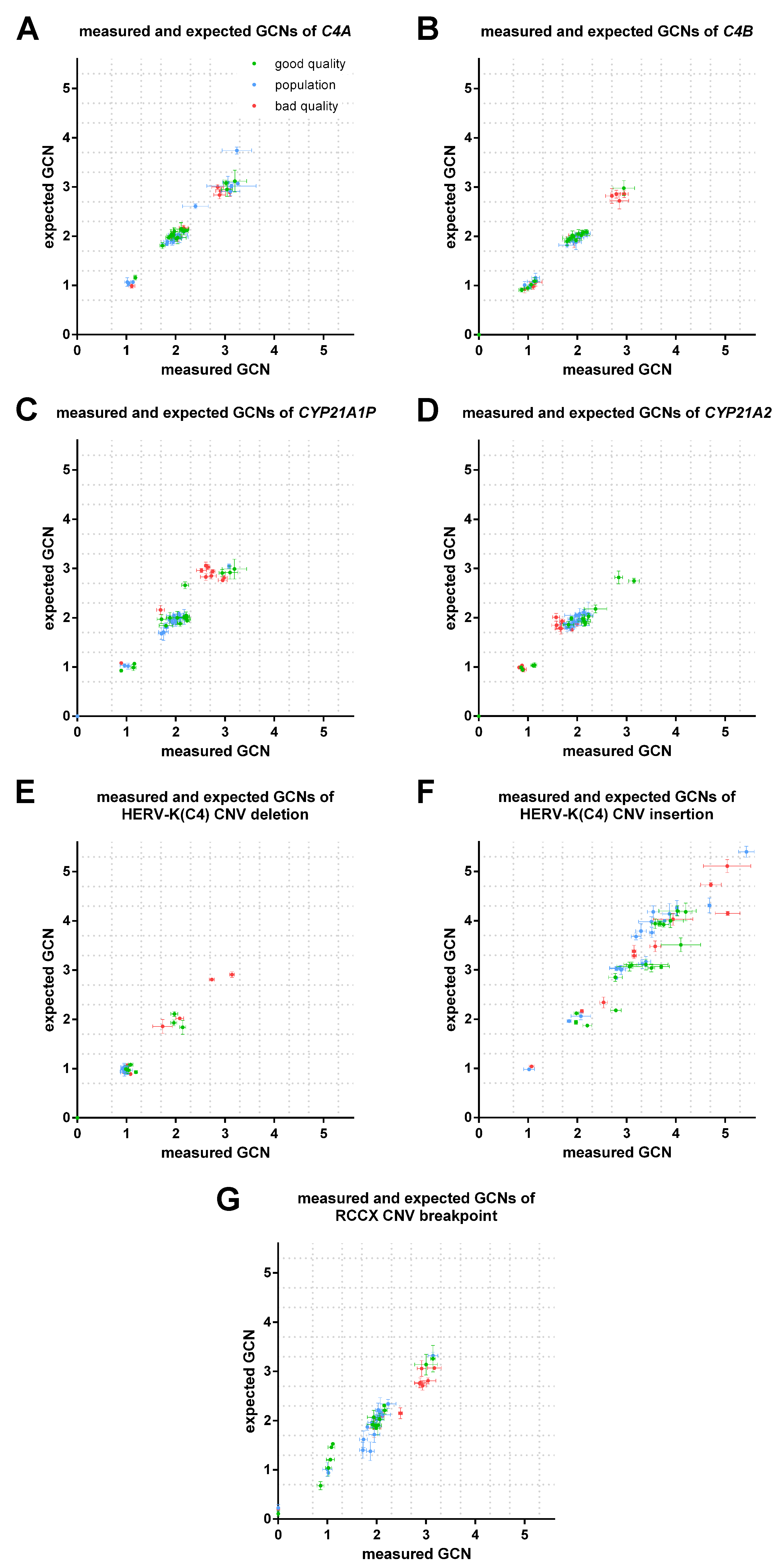

Supplement: S9 Fig — A multiple regression model was built from all measured GCNs of study groups based on the genomic relations. The expected total GCNs and RCCX CNV breakpoint GCN plus 2 were calculated in each replicate measurement based on the model. Then the expected average GCN of a particular target gene was calculated using the average of corresponding expected total GCNs in proportion to measured GCNs of the particular target genes and its allelic counterpart. (TIF) [file pone.0277299.s009.tif]

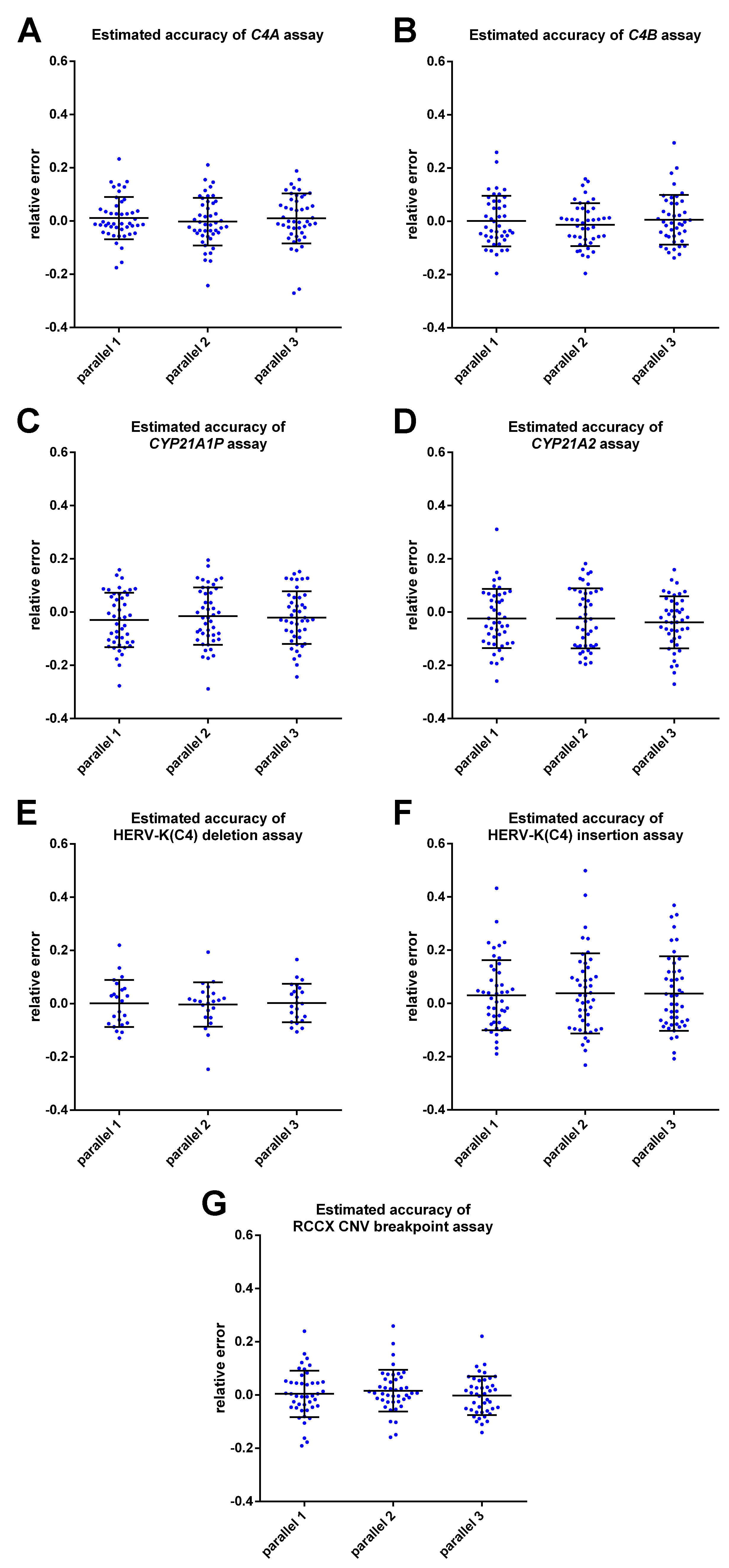

Supplement: S10 Fig — Estimated accuracy is expressed as the relative error of the qPCR measurements. Relative errors were not calculated for the samples with 0 GCN in the particular assay. Bars indicate means and standard deviation. (TIF) [file pone.0277299.s010.tif]

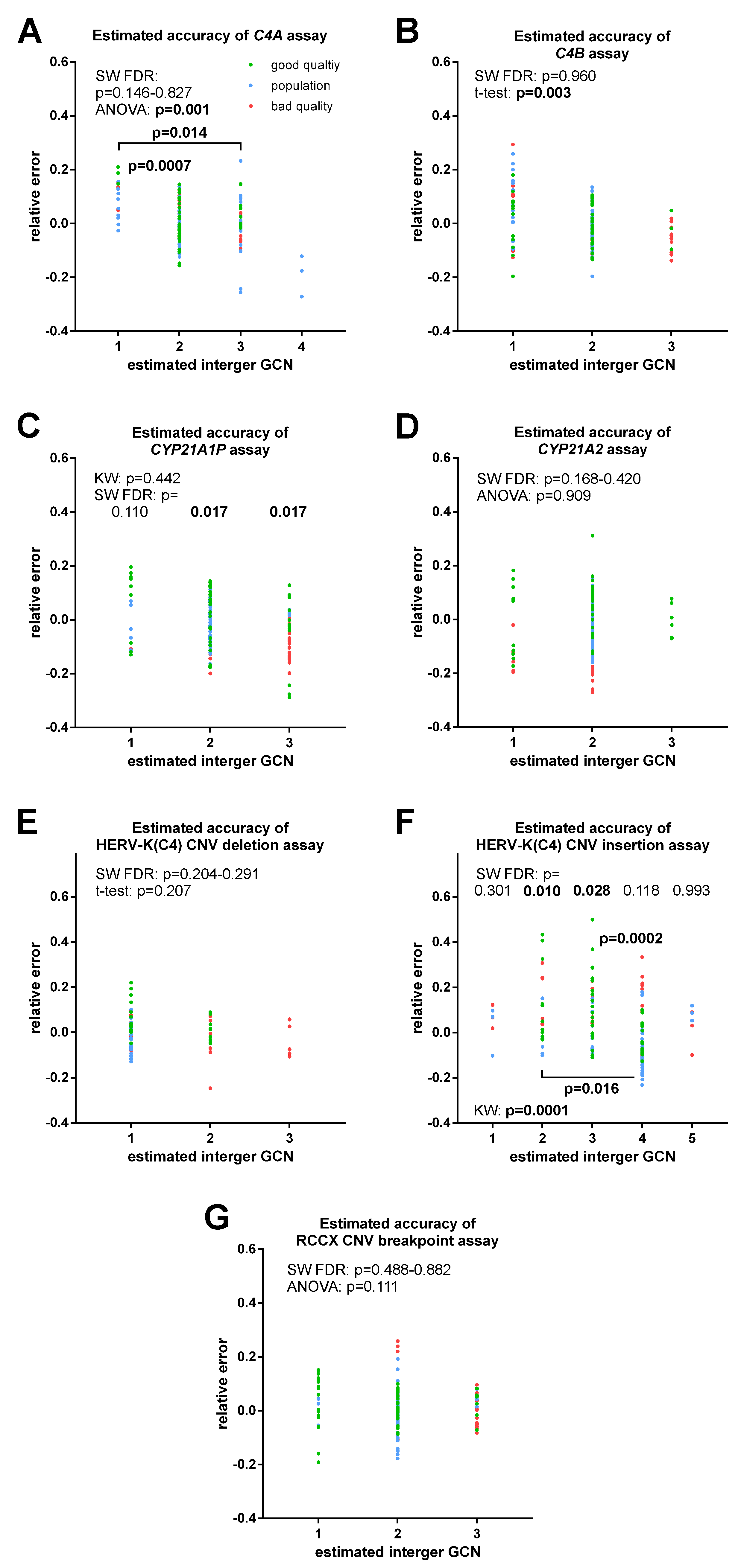

Supplement: S11 Fig — Estimated accuracy is expressed as the relative error of the qPCR measurements. Relative errors were not calculated for the samples with 0 GCN in the particular assay. Bars indicate means and standard deviation. All statistical tests were calculated based on “good quality” and “population” study groups. SW—Shapiro–Wilk test, FDR—false discovery rate method for multiple testing correction, KW—Kruskal-Wallis test. (TIF) [file pone.0277299.s011.tif]

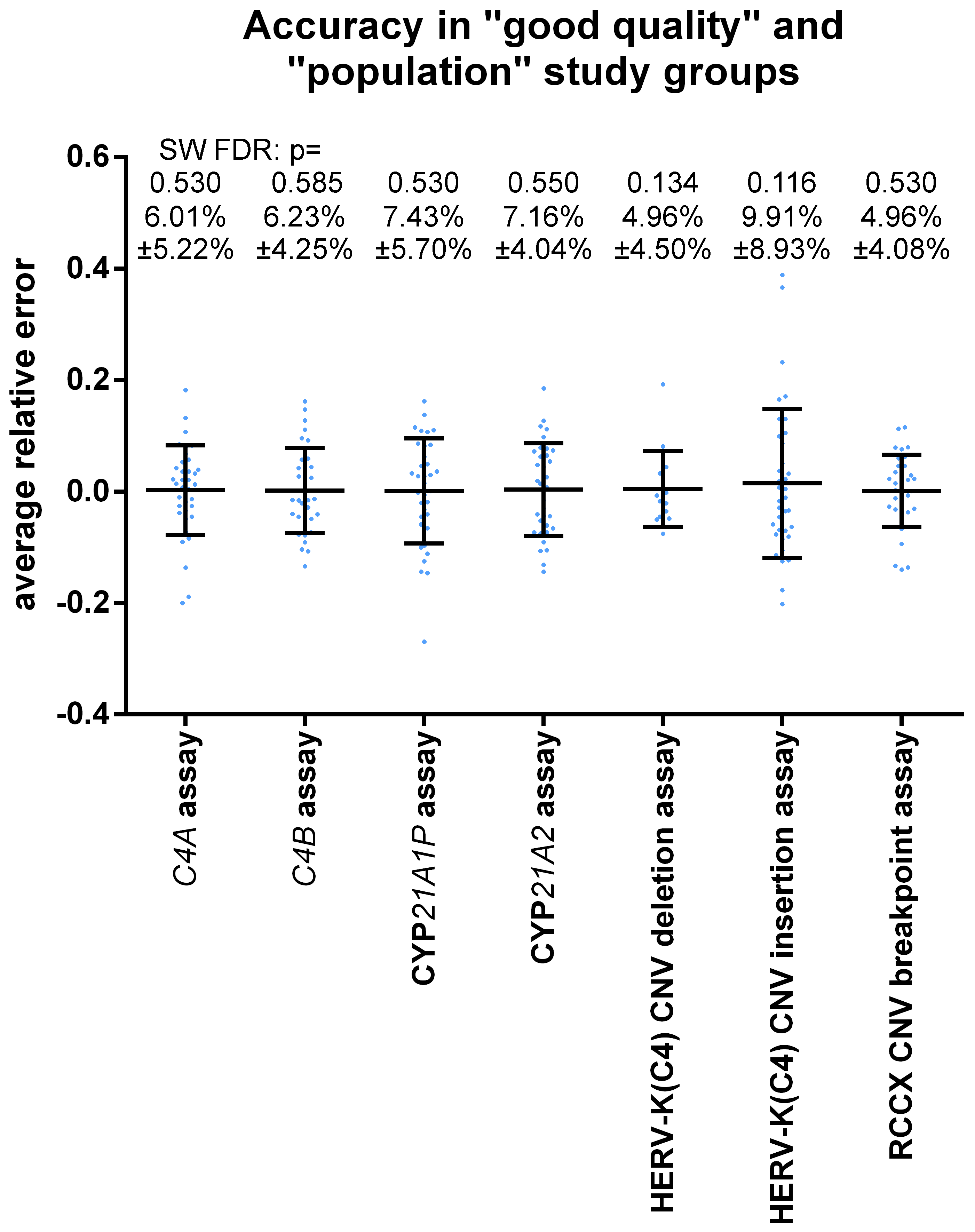

Supplement: S12 Fig — Estimated accuracy is expressed as the average relative error of the samples. The means and SDs of absolute values of average relative errors of the samples are indicated under the p-values of the Shapiro-Wilk test (SW). Relative errors were not calculated for the samples with 0 GCNs in the particular assay. Bars indicate means and standard deviation. FDR—false discovery rate method for multiple testing correction. The variances of average relative errors of samples in were significantly different (Levene’s test: p = 0.0026) between assays. However, only the difference between HERV-K(C4) CNV insertion and RCCX CNV breakpoint assays was significant (Levene’s test FDR: p = 0.04996) after multiple testing correction. (TIF) [file pone.0277299.s012.tif]

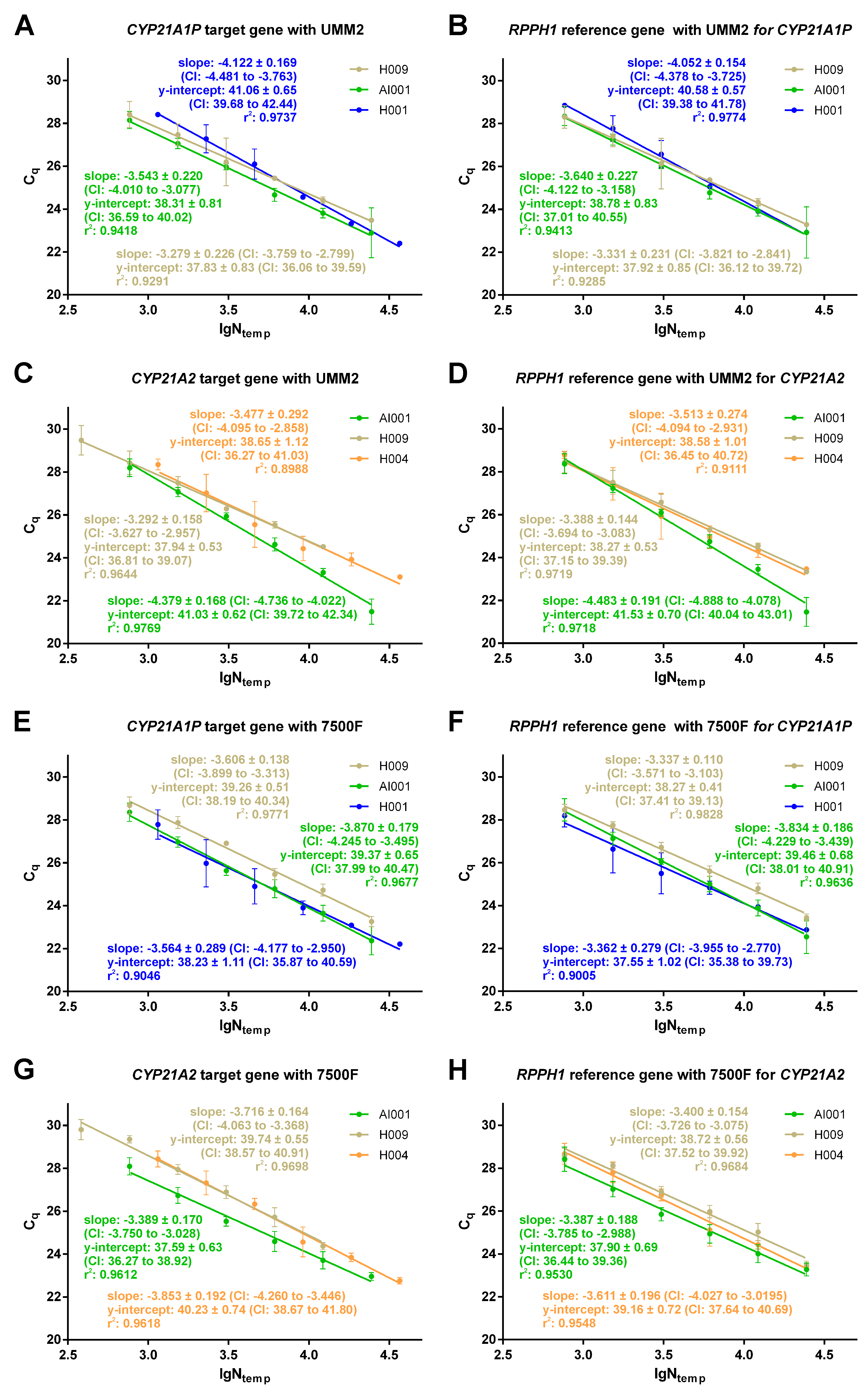

Supplement: S13 Fig — Ntemp is the copy number of the particular target genetic element in a measurement, which is conditional on the amount of genomic DNA in the series of dilutions (2.5, 5, 10, 20, 40 and 80 ng total DNA in a measurement) and the copy number of the particular genetic element in the diploid genome. CI means 95% confidence interval. UMM2—TaqMan universal master mix II, 7500F - 7500 Fast qPCR instrument. (TIF) [file pone.0277299.s013.tif]

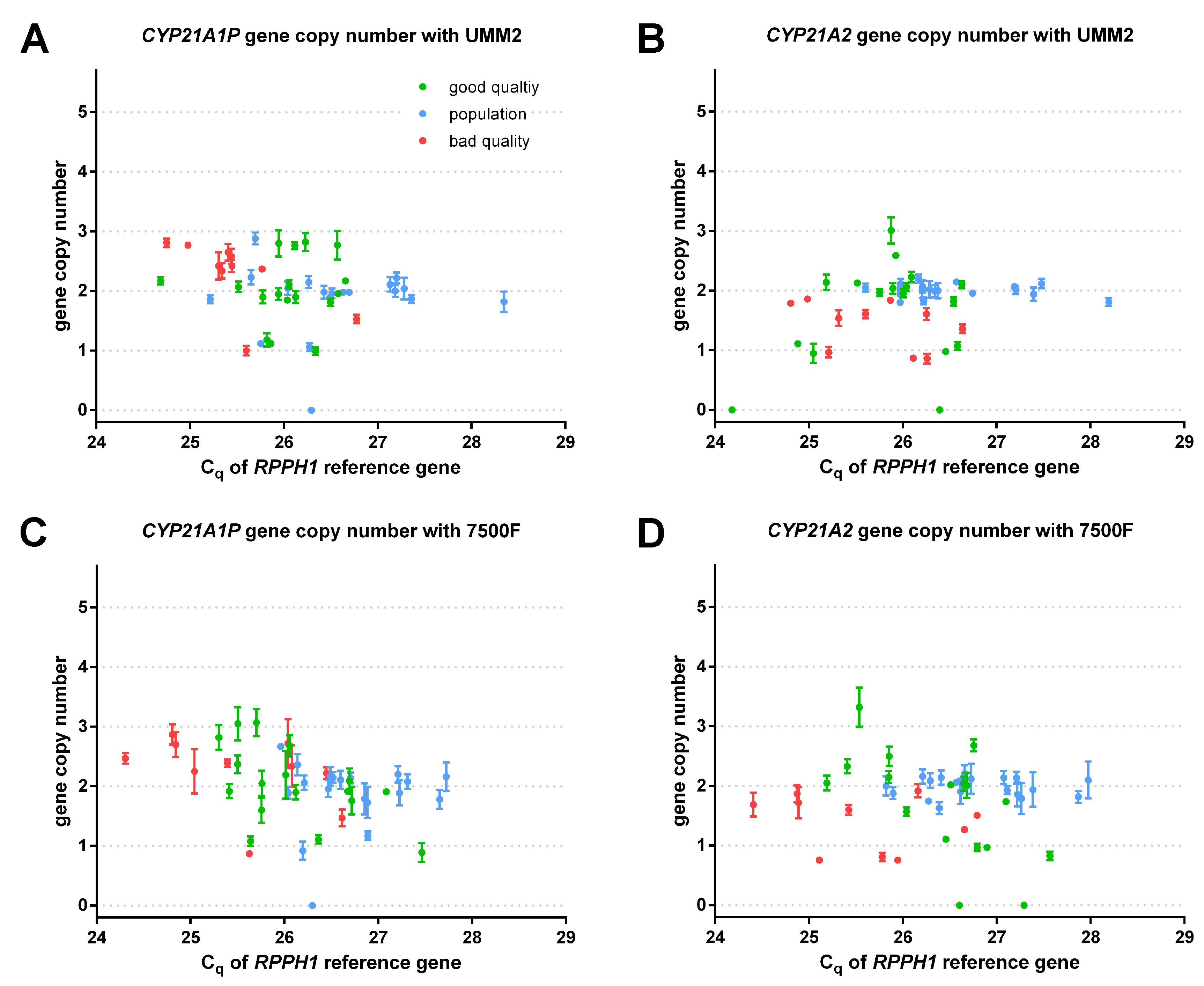

Supplement: S14 Fig — Bars indicate standard deviation. UMM2—TaqMan universal master mix II, 7500F - 7500 Fast qPCR instrument. (TIF) [file pone.0277299.s014.tif]

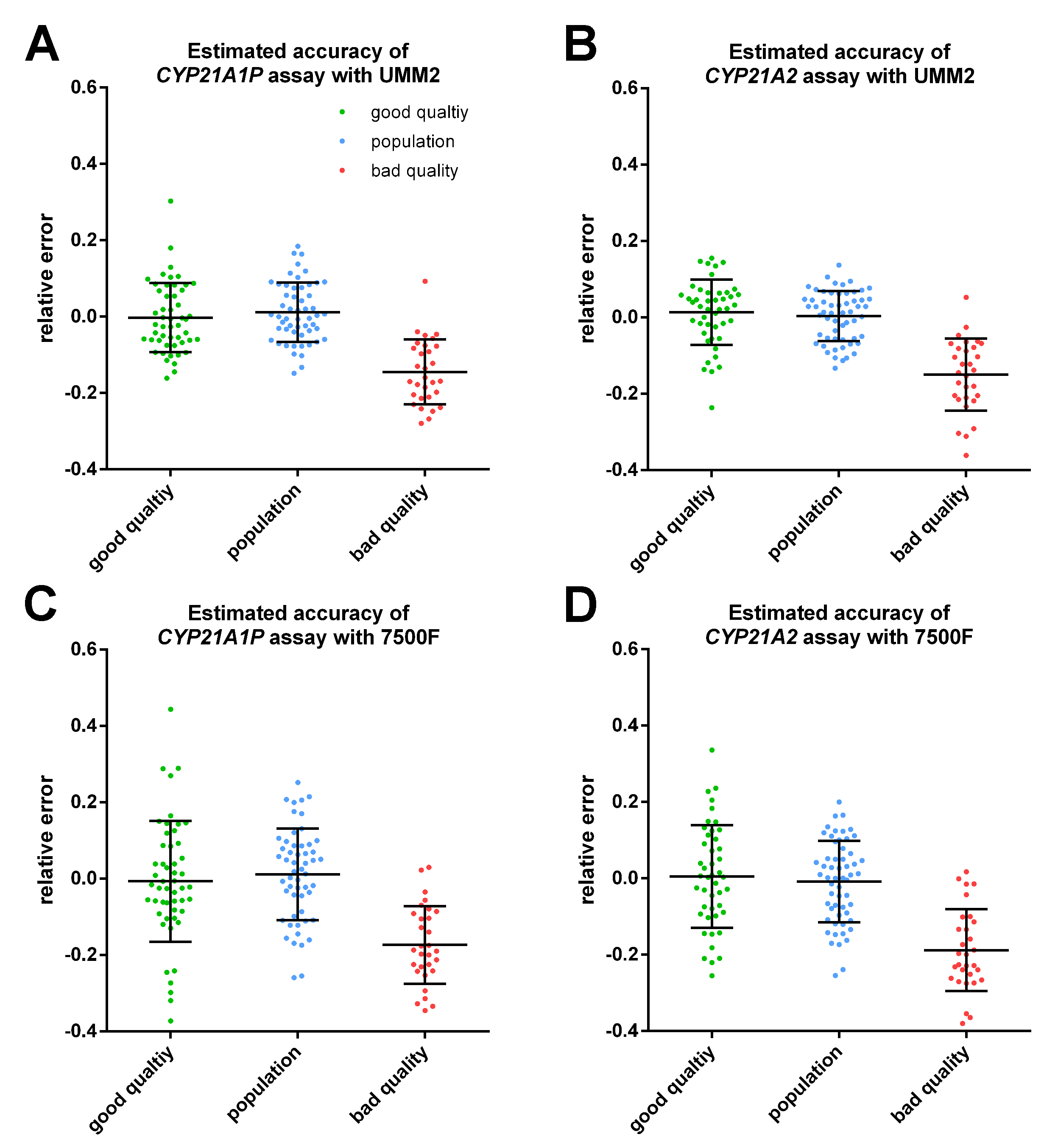

Supplement: S15 Fig — Estimated accuracy is expressed by the relative error of the qPCR measurements. Relative errors were not calculated for the samples with 0 GCN in the particular assay. Bars indicate means and standard deviation. UMM2—TaqMan universal master mix II, 7500F - 7500 Fast qPCR instrument. (TIF) [file pone.0277299.s015.tif]

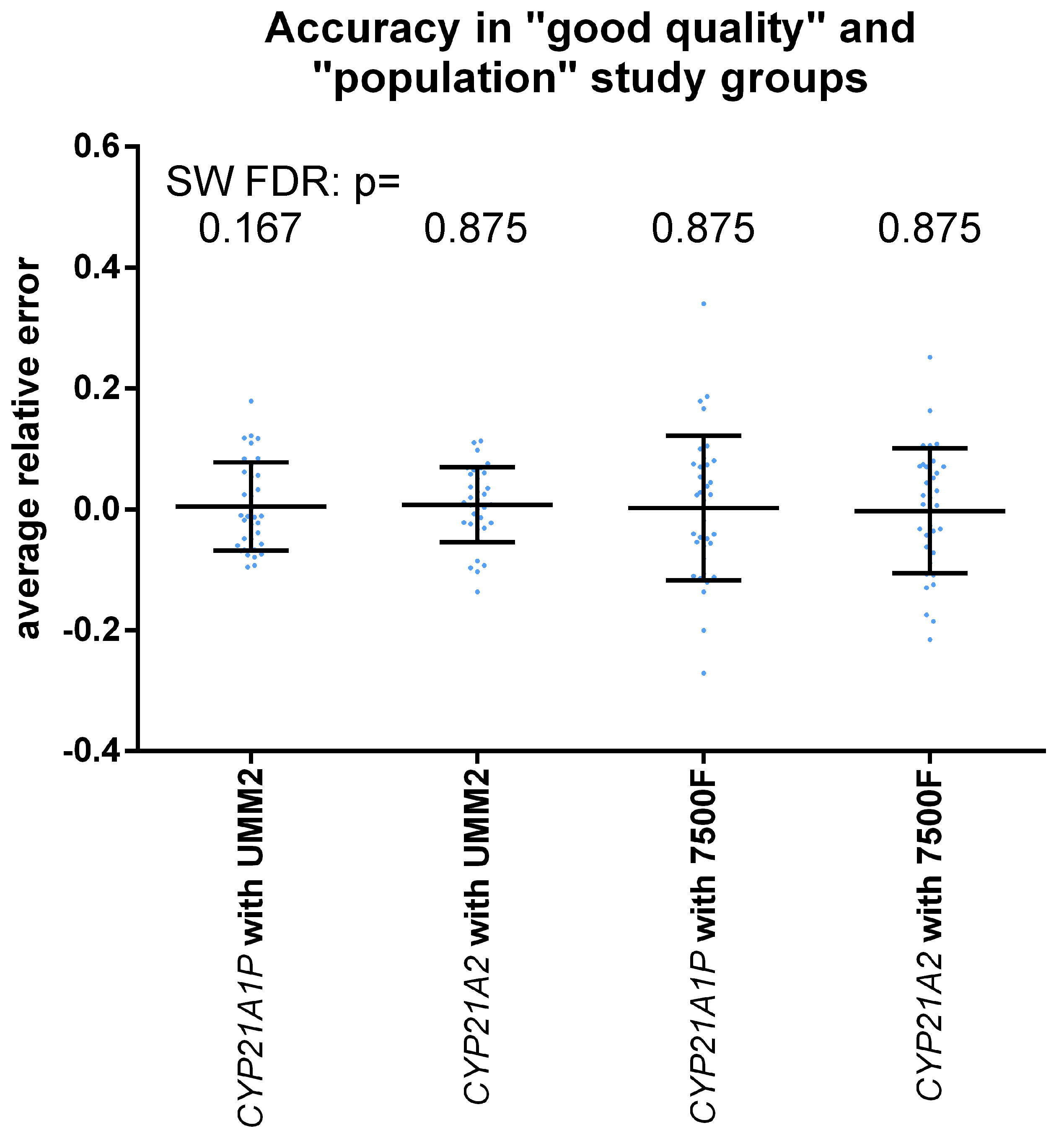

Supplement: S16 Fig — Estimated accuracy is expressed by the average relative error of the samples. Relative errors were not calculated for the samples with 0 GCN in the particular assay. Bars indicate means and standard deviation. SW—Shapiro–Wilk test, FDR—false discovery rate method for multiple testing correction. UMM2—TaqMan universal master mix II, 7500F - 7500 Fast qPCR instrument. (TIF) [file pone.0277299.s016.tif]

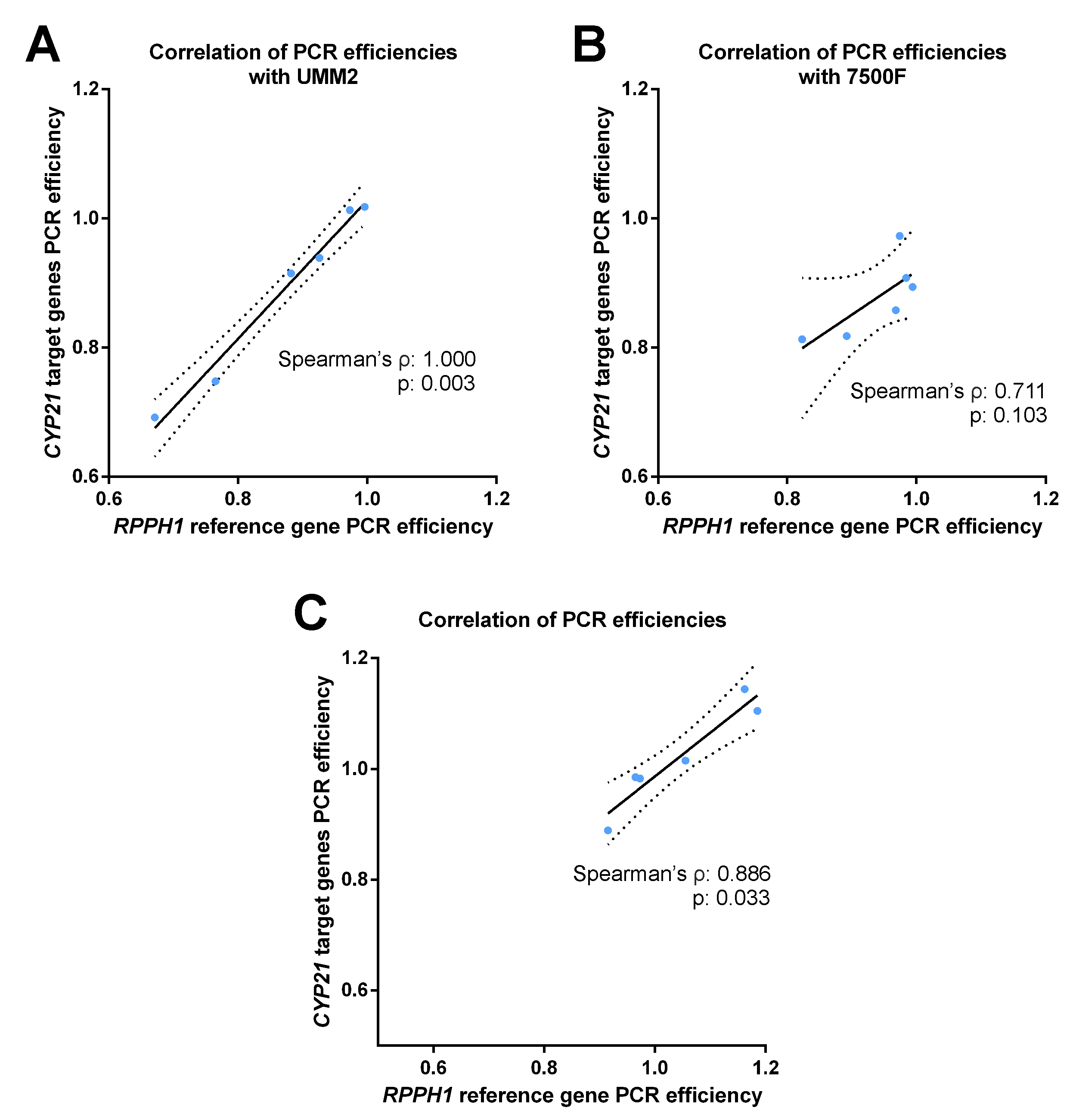

Supplement: S17 Fig — Correlations were made under the assumption that the PCR efficiencies of CYP21A1P and CYP21A2 assays behave in a similar way. Black line is a simple linear regression. Dotted line indicates the 95% confidence intervals. UMM2—TaqMan universal master mix II,7500F - 7500 Fast qPCR instrument. (TIF) [file pone.0277299.s017.tif]
