# Supplementary material for: Quantitative PCR from human genomic DNA: The determination of gene copy numbers for congenital adrenal hyperplasia and RCCX copy number variation
Source: PLoS One. 2022 Dec 1;17(12):e0277299. doi: 10.1371/journal.pone.0277299 (PMC9714944; doi:10.1371/journal.pone.0277299)
Supplement: S1 Table — There is no official consensus on the terms of ambiguity, concordance, misclassification and consistency, but they have been widely used in the literature of gene copy number determination. (PDF) [file pone.0277299.s018.pdf]

ambiguity	The state of a measured gene copy number not being close enough to an integer gene copy number to assign the measured gene copy number unambiguously to the integer gene copy number.
concordance	The state of an integer gene copy number estimated from measured gene copy number being identical to the genuine integer copy number.
consistency	The state of an integer gene copy number estimated from measured gene copy number being identical to another integer copy number, as expected.
copy number variation	A particular DNA segment occurs once in a haploid chromosome set (a haploid genome) as usual, but the same DNA segment (or very similar ones) may be repeated in a haploid chromosome set. If the number of repeats of a DNA segment varies between haploid chromosome sets, both numerical variants and genomic locus (or loci) of the repeated DNA segments are denoted by copy number variation.
gene copy number	GCN is the number of repeats of a gene in one or two sets of chromosomes. The vast majority of the genes occurs twice in a diploid genome, but the copy numbers of some genes can differ from two.
misclassification	The state of an integer gene copy number estimated from measured gene copy number not being identical to the genuine integer gene copy number.
paralogous gene variants	Paralogous genes (or gene variants) are the copies of the same gene (or very similar ones) with high DNA sequence similarity at the different loci of a haploid chromosome set.
HERV-K(C4) copy number variation	A deletion copy number variation derived from a virus, called human endogenous retrovirus K, insertion in the intron 9 if complement component 4 genes.
RCCX copy number variation	A tandem, complex, multi-allelic copy number variation on chromosome 6. It is named based on the old names of its genes, *RP1*-*C4*-*CYP21*-*TNXB*.
