# Supplementary material for: Quantitative PCR from human genomic DNA: The determination of gene copy numbers for congenital adrenal hyperplasia and RCCX copy number variation
Source: PLoS One. 2022 Dec 1;17(12):e0277299. doi: 10.1371/journal.pone.0277299 (PMC9714944; doi:10.1371/journal.pone.0277299)
Supplement: S2 Table — The term “accuracy” is used as a difference between a measured value and a “true” value determined by a reference material or method. “Ambiguity” is a state of a measured GCN not being close enough to an integer GCN to assign it clearly. “Misclassification” is a state of an integer GCN estimated from measured GCN not being identical to the genuine integer GCN. PMID–PubMed ID, avr–average, SD—standard deviation, ΔCq−Cq(target gene)-Cq(reference gene), CV%—coefficient of variation %. 1There are criteria, but no detailed information. 2It is implicitly stated on an graph. 3It is expressed in relation to the results of other method(s) for GCN determination. (PDF) [file pone.0277299.s019.pdf]

	DNA quality	primers & probes	analytical specificity	PCR efficiency	repeatability	reproducibility	accuracy	ambiguity	misclassification
Wu *et al.* 2007<br>PMID: 17709516	-	✓	-	-	avr GCN SD	-	-	-	-
Szabo *et al.* 2013<br>PMID: 24312389	-	✓	-	-	-	-	-	-	-
Szilagyi *et al.* 2006<br>PMID: 16403222	-	✓	-	✓	deviation in GCN	-	-	-	-
Parajes *et al.* 2007<br>PMID: 16403222	-	✓	-	✓	ΔC_q & GCN SD	ΔC_q & GCN SD	-	-	-
Leung *et al.* 2017<br>10.1373/jalm.2016.021923	(✓)[1]		-	-	-	-	-	-	✓
Fode *et al.* 2011<br>PMID: 21364933	-	✓	-	-	-	-	(✓)[2]	(✓)[2]	(✓)[3]
Perne *et al.* 2009<br>PMID: 20041854	(✓)[1]	✓	-	-	GCN SD & CV%	GCN CV%	-	(✓)[2]	(✓)[3]
Whale *et al.* 2012<br>PMID: 22373922	(✓)[1]	✓	✓	✓	-	-	-	-	-
Cantsilieris *et al.* 2014<br>PMID: 24885186	(✓)[1]	✓	-	-	-	-	-	(✓)[2]	(✓)[3]
