# Supplementary material for: Quantitative PCR from human genomic DNA: The determination of gene copy numbers for congenital adrenal hyperplasia and RCCX copy number variation
Source: PLoS One. 2022 Dec 1;17(12):e0277299. doi: 10.1371/journal.pone.0277299 (PMC9714944; doi:10.1371/journal.pone.0277299)
Supplement: S3 Table — (PDF) [file pone.0277299.s020.pdf]

Qiagen	Qiagen, Hilden, Germany
Roche	Roche, Penzberg, Germany
IHWG	International Histocompatibility Working Group, Fred Hutchinson Cancer Research Center, Seattle, Washington, USA.
5-Prime	5 Prime, San Francisco, CA, USA
Thermo Fisher Scientific	Thermo Fisher Scientific, Waltham, MA, USA
IDT	Integrated DNA Technologies, Coralville, Iowa, USA
Bioline	Bioline, London, UK
Agilent	Agilent, Santa Clara, CA, USA
MRC Holland	MRC Holland, Amsterdam, Netherlands
