# Supplementary material for: Quantitative PCR from human genomic DNA: The determination of gene copy numbers for congenital adrenal hyperplasia and RCCX copy number variation
Source: PLoS One. 2022 Dec 1;17(12):e0277299. doi: 10.1371/journal.pone.0277299 (PMC9714944; doi:10.1371/journal.pone.0277299)
Supplement: S4 Table — All primers and probes were purified by HPLC. At least one of the primers of a primer pair is bound to an intronic sequence. Allele-specific sites are indicated on the sequences by underscore. (PDF) [file pone.0277299.s021.pdf]

<table>
<tr><th>the name of quantitative PCR primers & probes</th><th>primer & probe sequences</th><th>length (bp)</th><th>position on NC_000006.12 reference sequence</th></tr>
<tr><td colspan="4">C4A & C4B target genes</td></tr>
<tr><td>C4_CN_F</td><td>GCAGGAGACATCTAACTGGCTTCT</td><td>24</td><td>31996024-31996047
32028762-32028785</td></tr>
<tr><td>C4_CN_R</td><td>CCGCACCTGCATGCTCCT</td><td>18</td><td>31996100-31996117
32028838-32028855</td></tr>
<tr><td>C4A_CN_probe</td><td>ACCCCTGTCCAGTGTTAG</td><td>18</td><td>31996080-31996097</td></tr>
<tr><td>C4B_CN_probe</td><td>ACCTCTCTCCAGTGATAC</td><td>18</td><td>32028818-32028835</td></tr>
<tr><td colspan="4">CYP21A1P & CYP21A2 target genes</td></tr>
<tr><td>CYP21A1P_F</td><td>ACCTGTCGTTGG|TCTCTGCTC</td><td>21</td><td>32006393-32006413</td></tr>
<tr><td>CYP21A2_F</td><td>GACCTGTCCTTGGGAGACTACT</td><td>22</td><td>32039120-32039141</td></tr>
<tr><td>CYP21_CN_R</td><td>CCAGCCTTACCTCACAGAACTC</td><td>22</td><td>32006501-32006522
32039237-32039258</td></tr>
<tr><td>CYP21_CN_probe</td><td>TGCTCCACCACTGGCTCCAT</td><td>20</td><td>32006471-32006490
32039207-32039226</td></tr>
<tr><td colspan="4">HERV-K(C4) CNV deletion & insertion target elements</td></tr>
<tr><td>C4Fin95</td><td>TTGCTCGTTCTGCTCATTCCTT</td><td>22</td><td>3265122-3265143 (NT_167245.2)
31984609-31984630
32017347-32017368</td></tr>
<tr><td>C4Sin9R-2 (deletion-specific primer)</td><td>GGCGCAGGCTGCTGTATT</td><td>18</td><td>3265207-3265224 (NT_167245.2)</td></tr>
<tr><td>C4L-3LTR-R (insertion-specific primer)</td><td>GTTGAGGCTGGTCCCCAACA</td><td>20</td><td>31984692-31984711
32017430-32017449</td></tr>
<tr><td>C4in95</td><td>CTCCTCCAGTGGACATG</td><td>17</td><td>3265146-3265162 (NT_167245.2)
31984633-31984649
32017371-32017387</td></tr>
<tr><td colspan="4">RCCX CNV breakpoint element</td></tr>
<tr><td>XA-RP2F2</td><td>TCCTGCAGTCATCTTTGTCTTCAG</td><td>24</td><td>32013236-32013259</td></tr>
<tr><td>XA-RP2R3</td><td>GAGCTGCAGATGGGATACCTTTAA</td><td>24</td><td>32013281-32013304</td></tr>
<tr><td>XA-RP2</td><td>CCAAATGCACAAGTACT</td><td>17</td><td>32013261-32013277</td></tr>
</table>
