# Supplementary material for: Quantitative PCR from human genomic DNA: The determination of gene copy numbers for congenital adrenal hyperplasia and RCCX copy number variation
Source: PLoS One. 2022 Dec 1;17(12):e0277299. doi: 10.1371/journal.pone.0277299 (PMC9714944; doi:10.1371/journal.pone.0277299)
Supplement: S5 Table — The RNaseP copy number (CN) reference assay contains the RPPH1 internal reference gene. A reaction usually contained 10 ng genomic DNA, but the reactions for calibration curves also contained 2.5, 5, 20, 40 or 80 ng genomic DNA. TaqMan fast advanced master mix was used as qPCR reagent with AmpliTaq™ Fast DNA Polymerase. Additional Mg2+, additional dNTP and other additives were not added to the reactions. MicroAmp fast 96-well reaction plates (cat. no.: 4346907) were used for qPCR measurements. (PDF) [file pone.0277299.s022.pdf]

	qPCR reagent	RNase P CN reference assay	target probe	target primers	total volume
C4A and *C4B* assays	1x	1x	0.12 μM	0.375 μM	10 μl
CYP21A1P, *CYP21A2*, HERV-K(C4) deletion, HERV-K(C4) insertion and RCCX CNV breakpoint assays			0.08 μM	0.25 μM	10 μl
2x total volume (*CYP21* assays)			0.08 μM	0.25 μM	20 μl
low primer concentration (*CYP21* assays)			0.08 μM	0.0625 μM	10 μl
low probe concentration (*CYP21* assays)			0.02 μM	0.25 μM	10 μl
