# Supplementary material for: Quantitative PCR from human genomic DNA: The determination of gene copy numbers for congenital adrenal hyperplasia and RCCX copy number variation
Source: PLoS One. 2022 Dec 1;17(12):e0277299. doi: 10.1371/journal.pone.0277299 (PMC9714944; doi:10.1371/journal.pone.0277299)
Supplement: S6 Table — m(gDNA)–mass of genomic DNA, Mnucl avr−average molar mass of nucleotide, Nnucl in hap gen−the amount of nucleotide pairs in the haploid human genome, NA−Avogadro’s number, CV–coefficient of variation, GCN–gene copy number, mGCNrep—measured gene copy number of replicate, Cq−quantification cycle, REmGCN−relative error of measured GCN of replicate, eiGCN–estimated integer gene copy number, NRMSE–normalized root-mean-square error. (PDF) [file pone.0277299.s023.pdf]

| clinical chemistry term | statistical metric | formula |
| --- | --- | --- |
| the amount of template ($N_{temp}$) | | $$N_{temp} = \frac{m(gDNA)}{2 \cdot M_{nucl\ avr} \cdot N_{nucl\ in\ hap\ gen}} \cdot N_A$$ |
| repeatability reproducibility (precision based on $C_q$) | pooled coefficient of variation % | $$CV\%_{pooled} = \frac{\sqrt{CV_1^2 + CV_2^2 + \cdots + CV_n^2}}{n} \cdot 100\%$$ |
| measured gene copy number of replicate | | $$mGCN_{rep} = 2 \cdot 2^{-(Cq(target) - Cq(ref))}$$ |
| average measured gene copy number of sample | | $$\overline{mGCN_{rep}}$$ |
| accuracy (based on GCN) | relative error of measurement | $$RE_{mGCN} = \frac{mGCN_{rep} - eiGCN}{eiGCN}$$ |
| | average relative error of sample | $$\overline{RE}_{mGCN}$$ |
| | normalized root-mean-square error of sample | $$NRMSE = \frac{\sqrt{\frac{\sum_{i=1}^{n}(-\left(\frac{\log_2(eiGCN)}{\log_2 2} - 1\right) - (Cq(target) - Cq(ref)))^2}{n}}}{\overline{Cq}(target)}$$ |
| | pooled NRMSE | $$NRMSE_{pooled} = \frac{\sqrt{NRMSE_1^2 + NRMSE_2^2 + \cdots + NRMSE_n^2}}{n}$$ |
