# Supplementary material for: Quantitative PCR from human genomic DNA: The determination of gene copy numbers for congenital adrenal hyperplasia and RCCX copy number variation
Source: PLoS One. 2022 Dec 1;17(12):e0277299. doi: 10.1371/journal.pone.0277299 (PMC9714944; doi:10.1371/journal.pone.0277299)
Supplement: S7 Table — Non-specific PCR product was observed only at the primer pairs of C4A and C4B target genes. (PDF) [file pone.0277299.s024.pdf]

		C4A target gene	*C4B* target gene	*CYP21A1P* target gene	*CYP21AA2* target gene	HERV-K(C4) CNV deletion target element	HERV-K(C4) CNV insertion target element	RCCX CNV breakpoint element
In silico data	forward primer length	24 bp		21 bp	22 bp	22 bp		24 bp
	reverse primer length	18 bp		22 bp		18 bp	20 bp	24 bp
	probe length	18 bp	18 bp	20 bp		17 bp		17 bp
	PCR product length	94 bp		130 bp	139 bp	103 bp	103 bp	69 bp
	GC content	60.6%	58.5%	60.8%	61.2%	53.4%	54.4%	43.5%
	melting temperature of PCR product	84.4 C°	83.6 C°	86.0 C°	86.4 C°	81.9 C°	82.3 C°	75.5 C°
	non-specific PCR product (Primer-Blast)	no	no	no	no	no	some weak	no
	secondary structure of PCR product (UNAFold)	no	no	no	no	no	no	no
Melting curve	melting temperature of specific PCR product	84.6 C°		86.5 C°	86.5 C°	83.3 C°	83.6 C°	78.3 C°
	melting temperature of non-specific PCR product	88.6 C°						
Micro-capillary electrophoresis	length of specific PCR product	101 bp	97 bp	131 bp	142 bp	106 bp	105 bp	74 bp
	concentration of specific PCR product	2.9 nM	6.7 nM	10.7 nM	2.3 nM	16.0 nM	4.0 nM	4.1 nM
	length of non-specific PCR product	118 bp	122 bp					
	concentration of non-specific PCR product	1.7 nM	2.4 nM					
