# Supplementary material for: Quantitative PCR from human genomic DNA: The determination of gene copy numbers for congenital adrenal hyperplasia and RCCX copy number variation
Source: PLoS One. 2022 Dec 1;17(12):e0277299. doi: 10.1371/journal.pone.0277299 (PMC9714944; doi:10.1371/journal.pone.0277299)
Supplement: S8 Table — Fixed effects were calculated based on all calibration curves of target genes or the RPPH1 gene, and a random effect of assays or samples were separately modeled. Both fixed effects of the target and RPPH1 reference genes were calculated from all assays, which can be interpreted as the estimated average slopes. This were very close to a perfect value of -3.322, corresponding to a PCR efficiency of 1. Individual random effects are expressed as a standard deviation from the fixed effect. (PDF) [file pone.0277299.s025.pdf]

		target genes	RPPH1 reference genes
fixed effect (lgN_{temp})		**-3.324**	**-3.310**
total random effect of assays		**0.055**	**0.045**
individual random effects by assays	*C4A* assay	-0.084	-0.063
	C4B assay	+0.002	-0.010
	CYP21A1P assay	-0.001	+0.008
	CYP21A2 assay	+0.064	+0.055
	HERV-K(C4) CNV deletion assay	+0.034	+0.012
	HERV-K(C4) CNV insertion assay	-0.058	-0.046
	RCCX CNV breakpoint assay	+0.042	+0.045
total random effect of DNA samples		**0.103**	**0.097**
individual random effects by DNA samples	AI001	-0.082	-0.072
	COX-QBL	+0.014	+0.003
	H001	-0.117	-0.112
	H004	+0.041	+0.048
	H009	+0.143	+0.132
