# Supplementary material for: Quantitative PCR from human genomic DNA: The determination of gene copy numbers for congenital adrenal hyperplasia and RCCX copy number variation
Source: PLoS One. 2022 Dec 1;17(12):e0277299. doi: 10.1371/journal.pone.0277299 (PMC9714944; doi:10.1371/journal.pone.0277299)
Supplement: S9 Table — The PCR efficiencies and estimated LOD were also assessed in the singleplex PCR reaction of each target or reference gene using the AI001 DNA sample. There were no significant differences between average PCR efficiencies from multiplex reactions (SW FDR: p = 0.857, ANOVA: p = 0.333, Tukey: p = 0.356–1.000 for target genes, SW FDR: p = 0.687–0.983, ANOVA: p = 0.604, Tukey: p = 0.627–1.000 for reference genes). LOD was estimated by the Hubaux-Vos method. SD—standard deviation, CI—confidence interval. (PDF) [file pone.0277299.s026.pdf]

			C4A assay	*C4B* assay	*CYP21A1P* assay	*CYP21A2* assay	HERV-K(C4) CNV deletion assay	HERV-K(C4) CNV insertion assay	RCCX CNV breakpoint assay
PCR efficiency	target gene	singleplex sample AI001	0.992	0.954	0.903	0.944	1.017	1.038	1.057
		sample AI001	0.974	0.978	0.983	0.985	1.024	1.002	1.011
		average ± SD	0.962 ± 0.056	1.000 ± 0.019	0.992 ± 0.108	1.048 ± 0.084	1.046 ± 0.020	1.065 ± 0.056	1.083 ± 0.077
		95% CI	0.823 - 1.101	0.951 - 1.048	0.723 - 1.262	0.839 - 1.257	0.996 - 1.097	0.925 - 1.204	0.892 - 1.274
	reference gene	singleplex sample AI001	0.996						
		sample AI001	0.944	0.953	0.973	0.965	0.984	0.972	0.985
		average ± SD	0.944 ± 0.066	1.000 ± 0.041	1.024 ± 0.142	1.061 ± 0.099	1.002 ± 0.024	0.975 ± 0.039	1.063 ± 0.113
		95% CI	0.780 - 1.108	0.897 - 1.103	0.671 - 1.377	0.815 - 1.306	0.943 - 1.061	0.878 - 1.071	0.782 - 1.344
estimated LOD	target gene	singleplex sample AI001	1.243	1.334	1.292	1.248	1.087	1.158	1.394
		average ± SD	1.42 ± 0.12	1.36 ± 0.09	1.47 ± 0.16	1.44 ± 0.12	1.43 ± 0.07	1.48 ± 0.06	1.43 ± 0.20
		95% CI	1.15 - 1.69	1.13 - 1.58	1.08 - 1.85	1.15 - 1.73	1.25 - 1.61	1.32 - 1.64	0.96 - 1.92
	reference gene	singleplex sample AI001	1.234						
		average ± SD	1.39 ± 0.13	1.35 ± 0.12	1.57 ± 0.42	1.52 ± 0.27	1.47 ± 0.07	1.34 ± 0.04	1.53 ± 0.35
