# Supplementary material for: Quantitative PCR from human genomic DNA: The determination of gene copy numbers for congenital adrenal hyperplasia and RCCX copy number variation
Source: PLoS One. 2022 Dec 1;17(12):e0277299. doi: 10.1371/journal.pone.0277299 (PMC9714944; doi:10.1371/journal.pone.0277299)
Supplement: S10 Table — All precisions are calculated by pooled coefficient of variation (CV) and expressed as CV%. Repeatability and reproducibility with same and different dilutions were assessed in positive control samples. The measurements of replicates for reproducibility were performed on different days. Reproducibility in “good quality”, “population” and “bad quality” study groups was assessed in samples with GCNs higher than zero. Some tendencies might be observed; the repeatability values from the measurement of the same dilutions tended to be lower than those from measurement of different dilutions, and repeatability values tended to be lower than reproducibility values. (PDF) [file pone.0277299.s027.pdf]

		C4A assay	*C4B* assay	*CYP21A1P* assay	*CYP21A2* assay	HERV-K(C4) CNV deletion assay	HERV-K(C4) CNV insertion assay	RCCX CNV breakpoint assay
target gene	repeatability same dilution	0.34	0.45	0.21	0.36	0.21	0.24	0.65
	reproducibility same dilution	0.62	0.41	0.40	0.63	0.48	0.38	1.01
	repeatability with dilution	0.59	0.48	0.52	0.27	0.35	0.40	0.53
	reproducibility with dilution	0.51	0.62	0.69	0.61	0.54	0.61	0.79
	reproducibility "good quality"	0.48	0.54	0.54	0.59	0.72	0.47	0.67
	reproducibility "population"	0.73	0.80	0.71	0.83	1.01	0.88	1.01
	reproducibility "bad quality"	0.97	0.56	0.66	0.58	0.42	0.48	0.68
reference gene	repeatability same dilution	0.30	0.24	0.18	0.26	0.28	0.21	0.62
	reproducibility same dilution	0.46	0.24	0.38	0.44	0.42	0.28	0.80
	repeatability with dilution	0.57	0.37	0.53	0.34	0.41	0.50	0.55
	reproducibility with dilution	0.46	0.55	0.65	0.62	0.60	0.66	0.76
	reproducibility "good quality"	0.50	0.51	0.59	0.50	0.60	0.54	0.75
	reproducibility "population"	0.80	0.82	0.70	0.89	0.85	0.93	0.95
	reproducibility "bad quality"	0.93	0.49	0.67	0.58	0.44	0.48	0.62
