# Supplementary material for: Quantitative PCR from human genomic DNA: The determination of gene copy numbers for congenital adrenal hyperplasia and RCCX copy number variation
Source: PLoS One. 2022 Dec 1;17(12):e0277299. doi: 10.1371/journal.pone.0277299 (PMC9714944; doi:10.1371/journal.pone.0277299)
Supplement: S11 Table — The measured GCNs between ±0.3 of an integer GCN were considered as unambiguous. (PDF) [file pone.0277299.s028.pdf]

	good quality	population	bad quality	total
C4A assay	17/17 (100%)	18/19 (95%)	10/10 (100%)	45/46 (98%)
C4B assay	17/17 (100%)	19/19 (100%)	10/10 (100%)	46/46 (100%)
CYP21A1P assay	17/17 (100%)	19/19 (100%)	5/10 (50%)	41/46 (89%)
CYP21A2 assay	16/17 (94%)	19/19 (100%)	5/10 (50%)	40/46 (87%)
HERV-K(C4) CNV deletion assay	17/17 (100%)	19/19 (100%)	10/10 (100%)	46/46 (100%)
HERV-K(C4) CNV insertion assay	12/17 (71%)	13/19 (68%)	8/10 (80%)	33/46 (72%)
RCCX CNV breakpoint assay	17/17 (100%)	19/19 (100%)	9/10 (90%)	45/46 (98%)
