# Supplementary material for: Quantitative PCR from human genomic DNA: The determination of gene copy numbers for congenital adrenal hyperplasia and RCCX copy number variation
Source: PLoS One. 2022 Dec 1;17(12):e0277299. doi: 10.1371/journal.pone.0277299 (PMC9714944; doi:10.1371/journal.pone.0277299)
Supplement: S12 Table — Concordance was assessed in the samples with unambiguous gene copy numbers (GCNs) compared to the GCNs from MLPA, Southern blot and array CGH or to the estimated integer GCNs. Percentages indicate the rate of correctly determined GCNs. Percentage is not calculated when n<9. (PDF) [file pone.0277299.s029.pdf]

		good quality	population	bad quality	total
C4A assay	to the data of other methods	1/1	15/16 (94%)		16/17 (94%)
	to estimated integer GCN	17/17 (100%)	17/18 (89%)	10/10 (100%)	44/45 (98%)
C4B assay	to the data of other methods	1/1	17/17 (100%)		18/18 (100%)
	to estimated integer GCN	17/17 (100%)	19/19 (100%)	10/10 (100%)	46/46 (100%)
CYP21A1P assay	to the data of other methods	15/16 (94%)	17/17 (100%)	3/3	35/36 (97%)
	to estimated integer GCN	16/17 (94%)	19/19 (100%)	5/5	40/41 (98%)
CYP21A2 assay	to the data of other methods	16/16 (100%)	18/18 (100%)	4/4	38/38 (100%)
	to estimated integer GCN	16/16 (100%)	19/19 (100%)	5/5	40/40 (100%)
HERV-K(C4) CNV deletion assay	to the data of other methods				
	to estimated integer GCN	17/17 (100%)	19/19 (100%)	10/10 (100%)	46/46 (100%)
HERV-K(C4) CNV insertion assay	to the data of other methods				
	to estimated integer GCN	10/12 (83%)	11/13 (85%)	7/8	28/33 (85%)
RCCX CNV breakpoint assay	to the data of other methods	16/16 (100%)	19/19 (100%)	6/6	41/41 (100%)
	to estimated integer GCN	17/17 (100%)	19/19 (100%)	9/9	45/45 (100%)
