# Supplementary material for: Quantitative PCR from human genomic DNA: The determination of gene copy numbers for congenital adrenal hyperplasia and RCCX copy number variation
Source: PLoS One. 2022 Dec 1;17(12):e0277299. doi: 10.1371/journal.pone.0277299 (PMC9714944; doi:10.1371/journal.pone.0277299)
Supplement: S13 Table — The peak heights of MLPA probes were determined by Coffalyser software with the default setting. All precisions are calculated by pooled coefficient of variation (CV) and expressed byas CV%. The precisions (repeatability and reproducibility) were assessed with the same dilutions of positive control samples in the same way as performed in qPCR assays. Peak heights equaling zero were excluded from calculations. (PDF) [file pone.0277299.s030.pdf]

length (bp)	MLPA hybridization probe	genomic position	repeatability	reproducibility
130	Reference probe 00797-L13645	5q	12.97	26.19
135	Reference probe 16316-L21434	3q	13.59	25.24
148	TNXB probe 19037-L14637	Exon 35	13.75	25.58
166	Reference probe 10729-L11311	6p	14.92	24.25
184	CYP21A1P probe 15221-L20262	Exon 3,del8bp location	14.18	24.11
190	CYP21A2 probe 15221-L20261	Exon 3, del8bp location	13.57	24.47
214	CYP21A2 probe 17261-L21169	Exon 7, F306+T location	13.70	22.28
220	CYP21A1P probe 17261-L21170	Exon 7, F306+T location	14.00	24.94
226	Reference probe 14471-L16191	4q	13.92	24.49
232	CYP21A2 probe 17270-L16990	V237E location Exon 6,	15.44	22.41
238	CYP21A2 probe 17271-L16989	Exon 6, M239K location	15.42	38.62
244	Reference probe 16307-L19696	13q	14.69	24.73
254	CYP21A2 probe 16645-L20231	Exon 3, I2G location, C-allele	20.40	29.12
259	CYP21A2 probe 16645-L20299	Exon 3, I2G location, A-allele	16.50	28.98
265	CYP21A2 probe 15220-L20667	Exon 4, I172N location	13.33	24.57
272	CYP21A1P probe 15220-L20668	Exon 4, I172N location	12.60	23.98
279	Reference probe 04988-L20303	8q	15.57	24.15
292	CYP21A1P probe 15945-L18079	Exon 1, -113 bp SNP	17.59	22.86
309	CYP21A2 probe 15944-L18351	Exon 1, -113 bp SNP	15.35	30.91
318	TNXB probe 15230-L14636	Exon 35	14.48	21.40
326	TNXB probe 19038-L17756	Exon 23	14.01	23.36
336	Reference probe 09027-L09281	1q	15.06	23.31
346	ATF6B probe 01979-L20800	Exon 1B	14.23	22.88
355	TNXB probe 15232-L01515	Exon 31	14.86	22.24
364	TNXB probe 15235-L04400	Exon 19	15.23	22.86
373	TNXB probe 15233-L15002	Exon 26	14.43	23.11
382	Reference probe 13329-L14755	18q	15.70	24.07
