# Supplementary material for: Quantitative PCR from human genomic DNA: The determination of gene copy numbers for congenital adrenal hyperplasia and RCCX copy number variation
Source: PLoS One. 2022 Dec 1;17(12):e0277299. doi: 10.1371/journal.pone.0277299 (PMC9714944; doi:10.1371/journal.pone.0277299)
Supplement: S14 Table — Estimations were done based on the means and variances of average relative errors of different GCNs. Estimation were not performed for GCNs with less than 5 samples. (PDF) [file pone.0277299.s031.pdf]

	C4A assay	*C4B* assay	*CYP21A1P* assay	*CYP21A2* assay	HERV-K(C4) CNV deletion assay	HERV-K(C4) CNV insertion assay	RCCX CNV breakpoint assay
ambiguity at 1 GCN		0.72%	1.32%	3.02%	>0.01%		0.03%
ambiguity at 2 GCN	1.37%	1.38%	7.04%	5.54%		41.13%	1.74%
ambiguity at 3 GCN	26.21%		47.24%			51.40%	
ambiguity at 4 GCN						50.73%	
misclassification at 1 GCN		>0.01%	>0.01%	>0.01%	>0.01%		>0.01%
misclassification at 2 GCN	>0.01%	>0.01%	>0.01%	0.01%		5.37%	>0.01%
misclassification at 3 GCN	0.89%		9.34%			12.42%	
misclassification at 4 GCN						11.35%	
