# Supplementary material for: Quantitative PCR from human genomic DNA: The determination of gene copy numbers for congenital adrenal hyperplasia and RCCX copy number variation
Source: PLoS One. 2022 Dec 1;17(12):e0277299. doi: 10.1371/journal.pone.0277299 (PMC9714944; doi:10.1371/journal.pone.0277299)
Supplement: S15 Table — The primers of CYP21A1P and CYP21A2 genes were tested in a LightCycler 1.0 instrument with Sybr Green dye, and several qPCR parameters such as total volume, annealing temperature, primer concentration, probe concentration, qPCR reagent (UMM2) and qPCR instrument (7500F) were changed in the FAMM-GS7 system usually used for the current study. All precisions are calculated by pooled coefficient of variation (CV) and expressed as CV%. Repeatability and reproducibility were assessed in positive control samples from the same dilution. FAMM—TaqMan fast advance master mix, UMM2—TaqMan universal master mix II, GS7—GeneStudio 7 qPCR instrument, 7500F - 7500 Fast qPCR instrument. (PDF) [file pone.0277299.s032.pdf]

		CYP21A1P target gene	*RPPH1* for *CYP21A1P* assay	*CYP21A2* target gene	*RPPH1* for *CYP21A2* assay
LightCycler 1.0 + Sybr Green	repeatability	0.64		0.34	
	reproducibility	0.88		1.09	
2x total volume (FAMM + GS7)	repeatability	0.35	0.31	0.26	0.37
	reproducibility	0.38	0.34	0.73	0.55
64 °C annealing T (FAMM + GS7)	repeatability	0.51	0.39	0.40	0.33
	reproducibility	0.54	0.45	0.39	0.30
lower primer concentration (FAMM + GS7)	repeatability	0.22	0.21	0.35	0.18
	reproducibility	0.15	0.08	0.43	0.31
lower probe concentration (FAMM + GS7)	repeatability	0.36	0.34	0.39	0.36
	reproducibility	0.33	0.40	0.63	0.34
UMM2 + GS7	repeatability	1.37	1.26	0.74	0.82
	reproducibility	1.35	1.18	1.23	1.20
FAMM + 7500F	repeatability	1.56	1.28	0.97	0.82
	reproducibility	2.65	2.45	1.10	0.88
