# Supplementary material for: Quantitative PCR from human genomic DNA: The determination of gene copy numbers for congenital adrenal hyperplasia and RCCX copy number variation
Source: PLoS One. 2022 Dec 1;17(12):e0277299. doi: 10.1371/journal.pone.0277299 (PMC9714944; doi:10.1371/journal.pone.0277299)
Supplement: S16 Table — SD—standard deviation, CI—confidence interval. UMM2—TaqMan universal master mix II, 7500F - 7500 Fast qPCR instrument. (PDF) [file pone.0277299.s033.pdf]

		CYP21A1P assay with UMM2	*CYP21A2* assay with UMM2	*CYP21A1P* assay with 7500F	*CYP21A2* assay with 7500F
target gene	sample AI001	0.915	0.692	0.813	0.823
	average ± SD	0.894 ± 0.136	0.881 ± 0.168	0.872 ± 0.051	0.883 ± 0.080
	95% CI	0.740 - 1.048	0.691 - 1.071	0.814 - 0.930	0.792 - 0.974
reference gene	sample AI001	0.882	0.671	0.973	0.974
	average ± SD	0.881 ± 0.116	0.857 ± 0.162	0.933 ± 0.096	0.945 ± 0.046
	95% CI	0.751 - 1.012	0.673 - 1.041	0.825 - 1.042	0.893 - 0.996
