# Supplementary material for: Quantitative PCR from human genomic DNA: The determination of gene copy numbers for congenital adrenal hyperplasia and RCCX copy number variation
Source: PLoS One. 2022 Dec 1;17(12):e0277299. doi: 10.1371/journal.pone.0277299 (PMC9714944; doi:10.1371/journal.pone.0277299)
Supplement: S17 Table — UMM2—TaqMan universal master mix II, 7500F - 7500 Fast qPCR instrument. (PDF) [file pone.0277299.s034.pdf]

	good quality	population	bad quality	total
CYP21A1P assay with UMM2	17/17 (100%)	19/19 (100%)	3/10 (30%)	39/46 (85%)
CYP21A2 assay with UMM2	16/17 (94%)	19/19 (100%)	6/10 (60%)	41/46 (89%)
CYP21A1P assay with 7500F	14/17 (82%)	17/19 (89%)	5/10 (50%)	36/46 (78%)
CYP21A2 assay with 7500F	13/17 (76%)	18/19 (95%)	7/10 (70%)	38/46 (83%)
