# Supplementary material for: Quantitative PCR from human genomic DNA: The determination of gene copy numbers for congenital adrenal hyperplasia and RCCX copy number variation
Source: PLoS One. 2022 Dec 1;17(12):e0277299. doi: 10.1371/journal.pone.0277299 (PMC9714944; doi:10.1371/journal.pone.0277299)
Supplement: S18 Table — Misclassifiaction was assessed in the samples with unambiguous GCNs compared to estimated integer GCNs. Percentage is not calculated for n<9. UMM2—TaqMan universal master mix II, 7500F - 7500 Fast qPCR instrument. (PDF) [file pone.0277299.s035.pdf]

	good quality	population	bad quality	total
CYP21A1P assay with UMM2	17/17 (100%)	19/19 (100%)	3/3	39/39 (100%)
CYP21A2 assay with UMM2	16/16 (100%)	19/19 (100%)	6/6	41/41 (100%)
CYP21A1P assay with 7500F	13/14 (93%)	17/17 (100%)	3/5	33/36 (92%)
CYP21A2 assay with 7500F	13/13 (100%)	18/18 (100%)	7/7	38/38 (100%)
