# Supplementary material for: Quantitative PCR from human genomic DNA: The determination of gene copy numbers for congenital adrenal hyperplasia and RCCX copy number variation
Source: PLoS One. 2022 Dec 1;17(12):e0277299. doi: 10.1371/journal.pone.0277299 (PMC9714944; doi:10.1371/journal.pone.0277299)
Supplement: S19 Table — The variances of average relative errors of samples in the “good quality” and “population” study groups of the assays with UMM2 and 7500F were significantly different. Top left cell of the table contains the test results for all four groups, other cells contains the results between pairs and after multiple testing correction by the false discovery rate method. UMM2—TaqMan universal master mix II, 7500F - 7500 Fast qPCR instrument. (PDF) [file pone.0277299.s036.pdf]

Levene's test: p=**0.0040**	*CYP21A2* assay with UMM2	*CYP21A1P* assay with 7500F	*CYP21A2* assay with 7500F
CYP21A1P assay with UMM2	0.1858	**0.0397**	0.1387
CYP21A2 assay with UMM2		**0.0092**	**0.0221**
CYP21A1P assay with 7500F			0.4489
