# Supplementary material for: Quantitative PCR from human genomic DNA: The determination of gene copy numbers for congenital adrenal hyperplasia and RCCX copy number variation
Source: PLoS One. 2022 Dec 1;17(12):e0277299. doi: 10.1371/journal.pone.0277299 (PMC9714944; doi:10.1371/journal.pone.0277299)
Supplement: S20 Table — Estimations were made based on the means and standard deviation of average relative errors. UMM2—TaqMan universal master mix II, 7500F - 7500 Fast qPCR instrument. (PDF) [file pone.0277299.s037.pdf]

	CYP21A1P assay with UMM2	*CYP21A2* assay with UMM2	*CYP21A1P* assay with 7500F	*CYP21A2* assay with 7500F
ambiguity at 1 GCN	>0.01%	>0.01%	1.24%	0.36%
ambiguity at 2 GCN	4.06%	1.66%	21.14%	14.54%
ambiguity at 3 GCN	17.23%	11.04%	40.47%	33.17%
ambiguity at 4 GCN	30.61%	23.12%	53.20%	46.66%
misclassification at 1 GCN	>0.01%	>0.01%	>0.01%	>0.01%
misclassification at 2 GCN	>0.01%	>0.01%	0.35%	0.07%
misclassification at 3 GCN	0.15%	0.02%	5.19%	2.35%
misclassification at 4 GCN	1.69%	0.52%	14.48%	8.94%
