# Supplementary material for: Quantitative PCR from human genomic DNA: The determination of gene copy numbers for congenital adrenal hyperplasia and RCCX copy number variation
Source: PLoS One. 2022 Dec 1;17(12):e0277299. doi: 10.1371/journal.pone.0277299 (PMC9714944; doi:10.1371/journal.pone.0277299)
Supplement: S21 Table — Pooled CV of target and reference gene (reproducibility or inter-assay precision), pooled NRMSD of target Cqs characterizing the normalization of Cqs of target genes by Cqs of the reference gene, and the standard deviation of the average relative error used for the estimation of ambiguity and misclassification rates are given for information purposes. Correlation was assessed by Spearman’s correlation. UMM2—TaqMan universal master mix II, 7500F - 7500 Fast qPCR instrument. (PDF) [file pone.0277299.s038.pdf]

		pooled CV, pooled NRMSE and SD of average relative error	CV of *RPPH1* C_q	NRMSE of C_qs	average relative error of GCN
CYP21A1P assay with UMM2	CV of target C_q	0.0327	**0.986** (p>0.0001)	0.307 (p=0.145)	0.239 (p=0.200)
	CV of *RPPH1* C_q	0.0317		0.280 (p=0.156)	0.220 (p=0.204)
	NRMSE of target C_qs	0.0045			**0.954** (p>0.0001)
	average relative error	**0.0398**			
CYP21A2 assay with UMM2	CV of target C_q	0.0277	**0.977** (p>0.0001)	-0.128 (p=0.525)	-0.287 (p=0.199)
	CV of *RPPH1* C_q	0.0272		-0.113 (p=0.525)	-0.250 (p=0.230)
	NRMSE of target C_qs	0.0042			**0.767** (p>0.0001)
	average relative error	**0.0376**			
CYP21A1P assay with 7500F	CV of target C_q	0.0185	**0.803** (p>0.0001)	-0.109 (p=0.640)	-0.162 (p=0.599)
	CV of *RPPH1* C_q	0.0176		-0.073 (p=0.678)	-0.147 (p=0.599)
	NRMSE of target C_qs	0.0078			**0.905** (p>0.0001)
	average relative error	**0.0722**			
CYP21A2 assay with 7500F	CV of target C_q	0.0160	**0.898** (p>0.0001)	-0.006 (p=0.975)	-0.177 (p=0.633)
	CV of *RPPH1* C_q	0.0158		0.093 (p=0.721)	-0.139 (p=0.652)
	NRMSE of target C_qs	0.0065			**0.824** (p>0.0001)
	average relative error	**0.0609**			
CYP21A1P assay with FAMM & GS7	CV of target C_q	0.0064	**0.747** (p>0.0001)	-0.146 (p=0.531)	-0.185 (p=0.531)
	CV of *RPPH1* C_q	0.0066		-0.110 (p=0.531)	-0.120 (p=0.531)
	NRMSE of target C_qs	0.0058			**0.910** (p>0.0001)
	average relative error	**0.0570**			
CYP21A2 assay with FAMM & GS7	CV of target C_q	0.0073	**0.811** (p>0.0001)	-0.059 (p=0.848)	-0.149 (p=0.803)
	CV of *RPPH1* C_q	0.0075		-0.099 (p=0.848)	-0.034 (p=0.848)
	NRMSE of target C_qs	0.0050			**0.842** (p>0.0001)
	average relative error	**0.0404**			
