# Supplementary material for: Quantitative PCR from human genomic DNA: The determination of gene copy numbers for congenital adrenal hyperplasia and RCCX copy number variation
Source: PLoS One. 2022 Dec 1;17(12):e0277299. doi: 10.1371/journal.pone.0277299 (PMC9714944; doi:10.1371/journal.pone.0277299)
Supplement: S22 Table — Pooled CV of target and reference gene (reproducibility or interassay precision), pooled NRMSD of target Cqs characterizing the normalization of Cqs of target genes by Cqs of reference gene, and the standard deviation of average relative error used for the estimation of ambiguity and misclassification rates are given for information purposes. Correlation was assessed by Spearman’s correlation. (PDF) [file pone.0277299.s039.pdf]

		pooled CV, pooled NRMSE and SD of average relative error	CV of *RPPH1* C_q	Normalized RMSE of C_qs	average relative error of GCN
C4A assay	CV of target C_q	0.0062	**0.715** (p>0.0001)	-0152. (p=0.377)	-0.187 (p=0.377)
	CV of *RPPH1* C_q	0.0068		-0.189 (p=0.377)	-0.160 (p=0.377)
	NRMSE of target C_qs	0.0052			**0.867** (p>0.0001)
	average relative error	**0.0522**			
C4B assay	CV of target C_q	0.0069	**0.809** (p>0.0001)	0.116 (p=0.781)	-0.027 (p=0.883)
	CV of *RPPH1* C_q	0.0070		0.126 (p=0.781)	0.072 (p=0.828)
	NRMSE of target C_qs	0.0047			**0.892** (p>0.0001)
	average relative error	**0.0425**			
HERV-K(C4) CNV deletion assay	CV of target C_q	0.0086	**0.700** (p=0.0076)	0.076 (p=0.974)	0.050 (p=0.974)
	CV of *RPPH1* C_q	0.0085		-0.041 (p=0.974)	-0.009 (p=0.974)
	NRMSE of target C_qs	0.0040			**0.829** (p>0.0001)
	average relative error	**0.0450**			
HERV-K(C4) CNV insertion assay	CV of target C_q	0.0072	**0.871** (p>0.0001)	0.055 (p=0.958)	-0.009 (p=0.958)
	CV of *RPPH1* C_q	0.0077		0.011 (p=0.958)	-0.069 (p=0.958)
	NRMSE of target C_qs	0.0075			**0.940** (p>0.0001)
	average relative error	**0.0893**			
RCCX CNV breakpoint assay	CV of target C_q	0.0087	**0.867** (p>0.0001)	-0.075 (p=0.968)	-0.023 (p=0.968)
	CV of *RPPH1* C_q	0.0088		-0.007 (p=0.968)	-0.100 (p=0.968)
	NRMSE of target C_qs	0.0041			**0.793** (p>0.0001)
	average relative error	**0.0408**			
